# Research progress on targeted regulatory proteins in the prevention and treatment of atherosclerosis

**DOI:** 10.3389/fimmu.2026.1791961

**Published:** 2026-07-01

**Authors:** Yuxin Wei, Fengmei Zhang, Yuanpeng Liao, Yan Zhao, Jiawei Guo

**Affiliations:** 1 Department of Vascular and Endovascular Surgery, The First Affiliated Hospital of Yangtze University, Jingzhou, Hubei, China; 2 Department of Pharmacology, School of Medicine, Yangtze University, Jingzhou, China

**Keywords:** apoptosis, atherosclerosis, epigenetics, HDACs, inflammation, KLFs, transcriptional regulation

## Abstract

Atherosclerosis (AS) is a chronic inflammatory disease characterized by lipid metabolic dysregulation and vascular cell phenotypic switching. Its progression is governed by intricate interactions between immune and vascular cells within multi-layered molecular networks. This review synthesizes recent evidence on the specific roles of key regulatory proteins in atherogenesis. We first examine how epigenetic modifiers, notably histone deacetylases, Sirtuins, EZH2, and TET2, remodel chromatin to activate pro-inflammatory gene programs. Subsequently, we delineate how critical transcription factors, such as KLFs, PPARs, Nrf2, and BACH1, couple metabolic homeostasis with anti-inflammatory responses. Furthermore, we detail how specific membrane signaling axes, including Eph receptors, Notch, and the SIRPα-CD47 checkpoint, alongside RIPK-mediated cell death pathways, govern endothelial dysfunction, macrophage polarization, and efferocytosis. We also discuss the contributions of UCP and ANGPTL proteins to cellular metabolism and inflammasome regulation. Notably, we propose an integrative perspective on cross-tier signaling crosstalk, highlighting complex feedback loops among histone deacetylases, KLFs, and Eph receptors that amplify vascular inflammation. Ultimately, comprehensively elucidating these immuno-vascular networks will pave the way for next-generation, precision-targeted therapies to mitigate the global burden of cardiovascular disease.

## 1 Introduction

Atherosclerosis (AS), a vascular pathology with chronic inflammation at its core, is a leading cause of cardiovascular-related morbidity and mortality worldwide. Contemporary evidence delineates AS as a chronic pathological process instigated by metabolic dysregulation and driven by immune inflammation, a process characterized by endothelial dysfunction, phenotypic switching of smooth muscle cells, and macrophage foam cell formation. Consequently, elucidating the deep-seated molecular regulatory networks spanning epigenetic regulation, transcriptional reprogramming, and membrane signal transduction is critical for identifying novel therapeutic targets.

At the cellular level, the interplay between epigenetic modifications and transcriptional networks governs disease progression. Epigenetic modifiers such as HDACs and TET2 remodel the chromatin landscape to license gene expression, whereas transcription factors including KLFs, PPARs, and Nrf2 function as signaling hubs that sense hemodynamic shear stress and oxidative stimuli to coordinate vascular anti-inflammatory defenses and metabolic homeostasis. Concurrently, membrane-associated signaling axes, specifically Eph receptors, the RIPK-mediated necroptosis pathway, and the CD47 immune checkpoint, profoundly modulate intercellular communication and plaque stability, while factors such as the UCP family and ANGPTL proteins further regulate local energy metabolism.

Notably, these hierarchical signaling modules do not operate in isolation but coalesce into a multidimensional interactive network. This review systematically synthesizes the key mechanisms spanning epigenetic regulation, transcriptional integration, and membrane signaling, with a particular focus on the synergistic crosstalk among these pathways. By mapping this comprehensive molecular regulatory landscape, we aim to provide a theoretical framework for deciphering AS pathogenesis and developing precision strategies for multi-target intervention.

## 2 Epigenetic regulation

### 2.1 HDACs in immune regulation and vascular remodeling

As the catalytic core of epigenetic machinery, the HDAC superfamily exerts divergent, isoform-specific effects on AS. Depending on the specific subtype and cellular microenvironment, HDAC members exert distinct pro-atherosclerotic or anti-atherosclerotic efficacy by precisely modulating target gene expression. Based on sequence homology, subcellular localization, and cofactor requirements, these enzymes are categorized into four distinct groups: Class I, Class II, Class III, and Class IV (1).

#### 2.1.1 HDAC3 in macrophage polarization and vascular senescence

**Figure 1:**
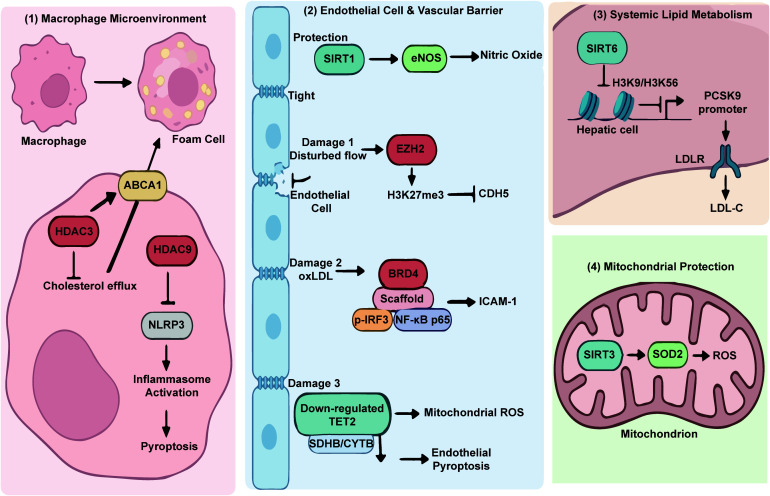
Epigenetic regulation of AS by HDACs (including sirtuins), EZH2, TET2, and BRD4. The progression of AS is tightly governed by a complex epigenetic network across distinct cellular microenvironments and systemic metabolic hubs. (1) Macrophage Microenvironment: Pro-atherosclerotic epigenetic drivers exacerbate lipid accumulation and inflammation. HDAC3 inhibits the cholesterol transporter ABCA1, impairing cholesterol efflux and driving foam cell formation. Concurrently, HDAC9 deacetylates NLRP3, triggering inflammasome activation and pyroptosis. (2) Endothelial Cell & Vascular Barrier: Endothelial homeostasis is regulated by a balance of protective and detrimental epigenetic modifiers. Protectively, SIRT1 activates eNOS to promote nitric oxide (NO) production. Conversely, disturbed flow upregulates EZH2, which utilizes H3K27me3 modifications to silence CDH5, thereby breaking endothelial tight junctions. Furthermore, oxLDL activates BRD4 to act as a scaffold for p-IRF3 and NF-κB p65, driving ICAM-1 expression; meanwhile, TET2 downregulation decreases SDHB/CYTB, leading to mitochondrial ROS accumulation and endothelial pyroptosis. (3) Mitochondrial Protection: Intracellularly, SIRT3 acts as a protective shield by activating SOD2 to scavenge ROS and prevent oxidative damage. (4) Systemic Lipid Metabolism: In the hepatic context, SIRT6 epigenetically suppresses the Pcsk9 promoter via H3K9/H3K56 modifications. This suppression decreases PCSK9 levels, preserving LDLR on the membrane and facilitating the systemic clearance of LDL-C. ABCA1, ATP-binding cassette transporter A1; AS, Atherosclerosis; BRD4, Bromodomain-containing protein 4; CDH5, Cadherin 5/VE-cadherin; CYTB, Cytochrome b; eNOS, Endothelial nitric oxide synthase; EZH2, Enhancer of zeste homolog 2; HDAC, Histone deacetylase; H3K27me3, Tri-methylation of lysine 27 on histone H3; H3K9, Lysine 9 on histone H3; H3K56, Lysine 56 on histone H3; ICAM-1, Intercellular adhesion molecule 1; LDL-C, Low-density lipoprotein cholesterol; LDLR, Low-density lipoprotein receptor; NF-κB, Nuclear factor kappa B; NLRP3, NLR family pyrin domain containing 3; NO, Nitric oxide; oxLDL, Oxidized low-density lipoprotein; PCSK9, Proprotein convertase subtilisin/kexin type 9; p-IRF3, Phosphorylated interferon regulatory factor 3; ROS, Reactive oxygen species; SDHB, Succinate dehydrogenase complex iron sulfur subunit B; SIRT/Sirtuins, Silent information regulator/Sirtuin; SOD2, Superoxide dismutase 2; TET2, Tet methylcytosine dioxygenase 2.

Within the Class I subfamily, HDAC3 exhibits the most intricate functional duality. Evidence indicates that in diabetic atherosclerotic plaques, aberrant upregulation of HDAC3 in macrophages exacerbates inflammation by driving M1 polarization and metabolic reprogramming. Mechanistically, HDAC3 localizes to the promoters of lipotoxic and inflammatory genes, where it modifies local chromatin architecture to sustain a glycolytic shift (2). Regarding lipid metabolism, HDAC3 downregulates the expression of the cholesterol transporter ABCA1, thereby inhibiting cholesterol efflux and facilitating foam cell formation (**Figure 1**) (3). Furthermore, in endothelial cells, HDAC3 overexpression potentiates endothelial-to-mesenchymal transition (EndMT) and inflammatory responses (4), collectively fueling the atherosclerotic process.

Conversely, emerging evidence highlights a protective dimension of HDAC3 signaling. Mechanistically, HDAC3 deacetylates histones to inhibit miR-19b transcription, relieving the suppression of PPARγ and subsequently promoting the ubiquitin-proteasomal degradation of NF-κB p65, which ultimately attenuates inflammation (5). In vascular smooth muscle cells (VSMCs), HDAC3 deficiency results in the accumulation of histone H4 lysine 12 lactylation, a modification that drives cellular senescence by epigenetically upregulating the senescence-associated secretory phenotype and restricting DNA repair capacity, accelerating AS progression (6). This protective potential is corroborated by clinical observations where HDAC3 expression is downregulated in peripheral blood mononuclear cells derived from patients with coronary artery disease (7).

#### 2.1.2 HDAC9 in inflammatory cascades and endothelial dysfunction

Among the Class II HDACs, HDAC9 stands out as a pivotal driver of AS, demonstrating a strong association with genetic risk factors and pathological mechanisms. Genetically, HDAC9 expression is governed by risk alleles; specifically, in myeloid cells, a non-coding cis-regulatory element harboring the lead risk variant rs2107595 directly propels high HDAC9 expression (8). Mechanistic studies further reveal that the rs2107595 variant abrogates the binding of the E2F3/Rb1 repressive complex to the promoter region, thereby promoting HDAC9 transcription (9).

Functionally, HDAC9 orchestrates a pro-atherogenic program via multiple synergistic pathways. In the context of inflammation, HDAC9 binds to and deacetylates IKKα/IKKβ, facilitating NF-κB p65 phosphorylation and nuclear translocation in concert with MAPK cascade activation, jointly serving as a transcriptional hub to amplify inflammatory gene expression (10). Simultaneously, serving as a direct upstream regulator of NLRP3, HDAC9 mediates NLRP3 deacetylation to trigger inflammasome activation and pyroptosis, thereby intensifying plaque inflammation (**Figure 1**) (8). Regarding vascular remodeling, HDAC9 drives EndMT and plaque instability by altering the acetylation status of specific histone residues H3K9 and H3K27, which epigenetically silences endothelial homeostatic genes and transcriptionally unleashes mesenchymal markers (10, 11).Notably, HDAC9 deficiency has been shown to resolve inflammation and enhance reverse cholesterol transport (12). Consistent with these findings, pharmacological inhibition using selective HDAC9 inhibitors, namely TMP195 and MC1568, effectively attenuates plaque burden and reinforces plaque stability in animal models (10, 11, 13), underscoring its therapeutic potential.

#### 2.1.3 Pleiotropic roles of other HDAC subtypes

Beyond these central actors, other HDAC isoforms fine-tune plaque progression, functioning primarily either as endothelial disruptors or context-dependent modulators. Specific isoforms, namely HDAC1, HDAC2, HDAC6, and HDAC11, predominantly exacerbate endothelial dysfunction and vascular inflammation. Specifically, HDAC1 and HDAC2 facilitate monocyte adhesion via STAT3 deacetylation and subsequent GATA6 hypermethylation (14), whereas HDAC6 profoundly impairs endothelial integrity by transcriptionally repressing VEGF and post-translationally deacetylating eNOS, which uncouples the enzyme and reduces nitric oxide synthesis (15, 16). Furthermore, the Class IV member HDAC11 acts as a potent pro-atherosclerotic driver by inducing endothelial pyroptosis and EndMT, alongside synergizing with DNMT3a to augment macrophage inflammation through a dual epigenetic repression mechanism involving coupled deacetylation and DNA hypermethylation of anti-inflammatory loci (17–19).

Conversely, distinct members of the HDAC family exert context-dependent or decidedly vasoprotective effects. In macrophages, HDAC2 alternatively acts as a stabilizer by collaborating with FOXO3 to repress Msr1, thereby limiting lipid uptake and necroptosis (20, 21) Likewise, HDAC4 restricts pathological macrophage metabolic reprogramming, albeit at the cost of promoting VSMC calcification (22). As crucial mechanosensitive protectors, HDAC5 and HDAC7 undergo phosphorylation-driven cytoplasmic translocation. This shift relieves the suppression of the KLF2/MEF2 complex, ultimately restoring transcriptional networks that safeguard vascular homeostasis. Likewise, HDAC4 restricts pathological macrophage metabolic reprogramming, albeit at the cost of promoting VSMC calcification As crucial mechanosensitive protectors, HDAC5 and HDAC7 undergo phosphorylation-driven cytoplasmic translocation. This shift relieves the suppression of the KLF2/MEF2 complex, ultimately restoring transcriptional networks that safeguard vascular homeostasis (23, 24).

### 2.2 Sirtuins in immuno-metabolic and vascular homeostasis

Classified as Class III HDACs, the Sirtuin family operates via NAD-dependent mechanisms that are distinct from other HDAC subtypes. SIRTs are universally recognized as protective bulwarks against vascular diseases, exerting beneficial effects across multiple cell types. Specifically, SIRT1 demonstrates anti-atherogenic and vasoprotective efficacy by suppressing the NF-κB signaling pathway to reduce macrophage foam cell formation (25) and deacetylating eNOS to promote endothelium-dependent vascular relaxation (26). Concurrently, SIRT3 maintains vascular homeostasis and mitigates oxidative stress under hypertensive conditions by deacetylating SOD2 to prevent mitochondrial ROS accumulation (27). Furthermore, SIRT6 suppresses hepatic lipid accumulation via PPARα activation to optimize systemic lipid metabolism (28), while locally protecting vascular smooth muscle cells from senescence to maintain plaque stability in atherosclerosis (29).

SIRTs maintain vascular integrity primarily by governing nitric oxide bioavailability, abrogating oxidative stress, and retarding endothelial senescence. SIRT1 directly potentiates vasodilation by deacetylating and activating eNOS to boost nitric oxide production (**Figure 1**) (26). Similarly, SIRT3 deacetylates and activates argininosuccinate synthase 1, the rate-limiting enzyme in the urea cycle, to enhance L-arginine biosynthesis and ensure sustained nitric oxide generation (30). Furthermore, functioning as the primary mitochondrial deacetylase, SIRT3 scavenges mitochondrial reactive oxygen species and prevents oxidative damage by activating superoxide dismutase 2 (**Figure 1**) (31, 32). PNotably, the vasculoprotective effects of PCSK9 inhibitors are partially mediated by SIRT3, which protects endothelial cells from inflammatory injury by mitigating mitochondrial reactive oxygen species accumulation, blocking NLRP3 inflammasome activation, and regulating autophagy (33). In the context of endothelial senescence, SIRT6 preserves cell cycle progression by maintaining the expression of the transcription factor FOXM1; consequently, its deficiency precipitates premature senescence and inflammatory responses (34). In addition to regulating transcription, SIRT6 directly deacetylates histone H3K9 to safeguard telomere integrity, thereby delaying endothelial aging and slowing AS progression (35). Meanwhile, SIRT3 promotes the clearance of damaged organelles through mitophagy to mitigate endothelial oxidative stress and arterial aging (32), though its direct impact on AS development appears limited (35).

Senescence, phenotypic switching, and calcification of VSMCs are critical drivers of plaque instability. SIRTs mitigate these pathological processes via NAD-dependent mechanisms. Research indicates that angiotensin II-induced VSMC senescence is closely linked to depleted intracellular NAD levels. Supplementation with NAD or inhibition of the NAD-consuming enzyme CD38 restores SIRT1 and SIRT3 activity, which subsequently revitalizes mitophagy and lysosomal function to block the secretion of senescence-associated small extracellular vesicles and prevent the propagation of senescence signals (36). Furthermore, the stability of SIRT6 is contingent upon the ubiquitin ligase CHIP; under hyperlipidemic conditions, CHIP downregulation accelerates SIRT6 degradation, leading to telomere damage and VSMC senescence (29). In diabetic environments, SIRT1 downregulation impairs DNA repair capacity. Restoring SIRT1 activity enhances the function of the ATM-MRN complex to repair high-glucose-induced DNA lesions, thereby inhibiting the transdifferentiation of VSMCs into osteoblast-like cells and delaying vascular calcification (37).

SIRTs further influence plaque stability by orchestrating macrophage polarization and inflammatory responses via a metabolic-inflammatory coupling mechanism. SIRT3 functions as a pivotal metabolic switch; in hypertension models, it maintains the activity of pyruvate dehydrogenase E1α by deacetylating lysine residue 83, thereby preventing the metabolic shift from oxidative phosphorylation to glycolysis. Loss of SIRT3 results in lactate accumulation and cellular acidification, which directly triggers NLRP3 inflammasome activation and IL-1β secretion, leading to perivascular adipose tissue dysfunction (38). Moreover, activation of the SIRT3-FOXO3α axis upregulates antioxidant enzymes including MnSOD to reduce reactive oxygen species levels, thereby blocking NLRP3 inflammasome assembly and inhibiting caspase-1-mediated macrophage pyroptosis (39). SIRTs also play a key role in transcriptional repression; specifically, SIRT1 alleviates vascular inflammation by deacetylating the NF-κB p65 subunit (40), while SIRT6 achieves this by deacetylating histone H3K9 at promoter regions (41), both leading to the suppressed transcription of pro-inflammatory cytokines such as TNF-α and IL-6.

Regarding lipid handling, SIRT6 attenuates foam cell formation by inducing autophagy and upregulating the cholesterol efflux transporters ABCA1 and ABCG1 (42). Similarly, defects in SIRT1 or SIRT3 signaling disrupt the PGC-1α-PPARα pathway by failing to deacetylate and activate these core transcriptional coactivators, leading to intracellular lipid accumulation (43). Beyond local vascular effects, SIRT6 improves the systemic lipid profile via epigenetic modifications in metabolic organs. Specifically, SIRT6 is recruited to the Pcsk9 promoter to inhibit expression through deacetylation of histone H3K9 and H3K56; the resulting downregulation of PCSK9 reduces LDL receptor degradation and promotes plasma LDL-cholesterol clearance (**Figure 1**) (42, 44). Additionally, SIRT6 represses the expression of SREBP1 and SREBP2, the master regulators of lipid synthesis, thereby reducing endogenous fatty acid and cholesterol production (44, 45).

### 2.3 EZH2 in immune reprogramming and vascular phenotypic switching

As the catalytic subunit of Polycomb Repressive Complex 2 (PRC2), EZH2 serves as a highly mechanosensitive mediator of AS initiation, primarily by compromising endothelial barrier integrity. In atheroprone regions characterized by disturbed flow, EZH2 is upregulated and directly represses the transcription of CDH5 via histone H3 lysine 27 trimethylation (H3K27me3). This epigenetic silencing disrupts endothelial junctions and increases permeability (**Figure 1**), thereby facilitating subendothelial lipid retention and leukocyte infiltration (46), Conversely, pharmacological inhibition or genetic knockdown of EZH2 restores endothelial integrity by diminishing H3K27me3 levels and relieving the repression of SOCS3. The subsequent upregulation of SOCS3 blockades central inflammatory hubs, particularly the NF-κB and STAT signaling networks, effectively suppressing the osteogenic differentiation and calcification of valvular interstitial cells (47). Additionally, downregulating EZH2 can restore endothelial integrity by increasing the expression of junctional adhesion molecules critical for the vascular barrier, such as VE-cadherin and PECAM-1 (46). Thus, targeting EZH2 represents a promising strategy for reestablishing vascular homeostasis.

During plaque progression, EZH2 remodels the functional landscape of macrophages and neutrophils across three distinct dimensions: lipid metabolism, inflammatory signaling, and cell motility.Regarding lipid handling, EZH2 acts as a critical driver of foam cell formation. The long non-coding RNA GAS5, which is enriched in plaques, recruits EZH2 to the ABCA1 promoter to epigenetically silence its transcription via H3K27me3, thereby impeding cholesterol efflux and accelerating lipid accumulation (48). In the context of inflammation, EZH2 mediates the transcriptional repression of Socs3 through H3K27me3; this down-regulation of Socs3 relieves its inhibition on TRAF6, thereby unleashing MyD88-dependent NF-κB signaling and augmenting the secretion of proinflammatory cytokines such as IL-6 and IL-12 (49). In terms of cell movement, beyond its canonical histone methyltransferase activity, EZH2 exerts non-canonical effects in the cytoplasm by methylating the cytoskeletal protein Talin1. This modification modulates integrin signaling and has been shown to enhance the migration of neutrophils, thereby exacerbating plaque inflammation (49).

EZH2 further propels AS by orchestrating VSMCs phenotypic switching and fibrosis, key processes underlying plaque vulnerability and remodeling. Following vascular injury or PDGF-BB stimulation, the circular RNA circHECTD1 interacts with KHDRBS3 to stabilize EZH2 mRNA. The resulting elevation in EZH2 expression silences the contractile marker SM-22α, promoting VSMC proliferation, migration, and neointimal hyperplasia (50). Additionally, the PCSK9-induced lncRNA SNHG16 recruits EZH2 to epigenetically repress Tumor Necrosis Factor Receptor-Associated Factor 5 (TRAF5), facilitating aberrant VSMC proliferation and foam cell formation (51), whereas EZH2-mediated downregulation of miR-139-5p activates the STAT1 pathway to disrupt endothelial tube formation and inhibit physiological angiogenesis (52). Under hypoxic conditions, the CDK2/Cyclin A complex phosphorylates and activates EZH2, which subsequently silences the mitochondrial protein Poldip2. This triggers a metabolic shift toward glycolysis and the hexosamine biosynthesis pathway, leading to proteasome inhibition, stabilization of the transcription factor SRF, and upregulation of the pro-fibrotic mediator CCN2 (CTGF), ultimately aggravating vascular fibrosis (53).

In the adaptive immune compartment, EZH2 promotes disease progression by restricting the protective differentiation of CD4+ T cells. High EZH2 expression in plaque-resident T cells directly represses the transcription factor Zbtb16 and the cytokine IL-4 via H3K27me3 marks. This suppression impairs the induction of anti-inflammatory macrophage polarization, thereby sustaining a high plaque burden (54).

### 2.4 TET2 in clonal hematopoiesis and vascular integrity

TET2 is an α-ketoglutarate- and ferrous iron-dependent dioxygenase that historically mediates DNA demethylation by catalyzing the sequential oxidation of 5-methylcytosine (55). Beyond its established role in hematopoietic development, TET2 exerts crucial epigenetic control in maintaining innate immune homeostasis and facilitating inflammation resolution by actively repressing pro-inflammatory gene transcription (56).

TET2 is a dioxygenase dependent on α-ketoglutarate and ferrous iron, functioning primarily to mediate DNA demethylation by catalyzing the sequential oxidation of 5-methylcytosine. Beyond its established role in hematopoietic development, TET2 exerts crucial epigenetic control in maintaining innate immune homeostasis and facilitating inflammation resolution (57).

Somatic mutations in the TET2 gene induce a phenomenon termed clonal hematopoiesis of indeterminate potential (CHIP), which has emerged as a central driver of AS. The pathogenic impact of clonal hematopoiesis in atherosclerotic cardiovascular disease persists across populations with varying low-density lipoprotein cholesterol levels, indicating that it drives disease primarily through non-lipid metabolic pathways (58). Direct analysis of plaques from patients with peripheral artery disease confirms that immune cells derived from TET2-mutant clones actively participate in plaque architecture (59). Moreover, pathological conditions such as hyperlipidemia induce excessive proliferation of hematopoietic stem cells, establishing a positive feedback loop that perpetuates disease progression (60). Clinical studies further demonstrate that TET2 mutation-driven clonal expansion is an independent predictor of significantly increased coronary artery disease risk and cardiovascular mortality, an association that remains robust after adjustment for traditional risk factors (61, 62).

In myeloid cells and macrophages, TET2 deficiency triggers robust proinflammatory responses via a multidimensional signaling network, significantly accelerating plaque progression. Epigenetically, TET2 loss induces hypermethylation of the Dusp10 promoter, suppressing its expression and leading to sustained JNK1 activation. This cascade drives the deubiquitinating enzyme BRCC3 and its scaffold protein ABRO1 to deubiquitinate NLRP3, thereby enhancing inflammasome activity and resulting in massive IL-1β release and the formation of neutrophil extracellular traps (63). Furthermore, TET2 deficiency significantly upregulates IL-6 expression in macrophages. This inflammatory factor subsequently activates the PI3K-AKT pathway to induce high expression of the colony-stimulating factor 1 receptor, which not only confers enhanced anti-apoptotic capacity to mutant macrophages—promoting their accumulation—but also amplifies systemic monocyte recruitment to lesion sites (64). TET2 deficiency can also induce type I interferon responses by failing to resolve endogenous DNA damage, which activates the cGAS-STING sensing pathway to drive broad transcriptional amplification of inflammatory genes (65). Ultimately, these mechanisms profoundly reshape the cellular composition of plaques. TET2-deficient macrophages exhibit a unique transcriptional profile characterized by tissue-resident markers and high secretion of chemokines such as Cxcl1 and Ccl2 (66). In the context of TET2/Jak2 co-mutations, IL-1β-mediated signaling further suppresses the accumulation of protective fibroblast-like cells within plaques, directly causing fibrous cap thinning and increasing rupture risk (67).

Beyond the hematopoietic system, endothelial TET2 homeostasis crucially suppresses atherogenesis. Upstream microenvironmental cues, encompassing low shear stress and the dysbiotic gut microbiota-derived metabolite trimethylamine N-oxide (TMAO), directly disrupt vascular epigenetic networks. Specifically, these pathogenic stimuli downregulate the DNA demethylase TET2 and its downstream targets SDHB and CYTB (**Figure 1**). Consequently, this epigenetic suppression provokes mitochondrial reactive oxygen species accumulation, ultimately driving endothelial pyroptosis and exacerbating vascular injury (68, 69).

In VSMCs, TET2 serves as a core regulator of the contractile phenotype, with its expression finely tuned by a non-coding RNA network. The circular RNA circMAP3K5 functions as a sponge for miR-22-3p, relieving the repression of TET2 to sustain VSMC differentiation and suppress neointimal hyperplasia (70). Intercellular communication also plays a role; endothelial cells transfer TET2 protein to VSMCs via exosomes, whereas activation of CD137 signaling disrupts this balance, leading to uncontrolled phenotypic switching and neointimal formation (71). Notably, the TET2-mutant microenvironment in aged bone marrow profoundly alters the clonal expansion patterns of smooth muscle-derived cells, holistically reshaping plaque architecture to exacerbate progression and diminish stability (72).

### 2.5 BRD4 in immune activation and vascular senescence

As a prominent member of the Bromodomain and Extraterminal protein family, BRD4 functions as an epigenetic reader that assembles transcriptional complexes to instigate pathogenic gene programs. Mechanistically, BRD4 relies on its bromodomains to specifically recognize acetylated lysine residues on chromatin, including histone H3 lysine 27 acetylation and histone H3 lysine 9 acetylation. This process targets inflammatory genes to induce cytokine expression while concurrently suppressing autophagy, thereby synergistically driving the progression of AS. This mechanism is critically dependent on the activity of histone acetyltransferase P300. Stimulation by oxidized low-density lipoprotein or reactive oxygen species amplifies P300 activity, which catalyzes histone acetylation and subsequently recruits BRD4 to chromatin, forming a self-reinforcing “stimulation-acetylation-BRD4 recruitment” loop (73). Furthermore, at the promoter regions of inflammatory genes, BRD4 colocalizes with the mediator MED1 and transcription factors such as NF-κB and IRF3 to form membrane-less, phase-separated transcriptional condensates. These structures highly concentrate transcriptional machinery like RNA polymerase II, triggering an explosive transcriptional burst of inflammatory mediators including IL-6, IL-8, and ICAM-1 (73, 74).

In the vascular wall, BRD4 propels AS through distinct synergistic mechanisms across multiple cell types. In endothelial cells, BRD4 integrates various inflammatory cascades to mediate the expression of pro-atherogenic leukocyte-tethering adhesion molecules, specifically VCAM-1, ICAM-1, and E-selectin, thereby driving targeted leukocyte recruitment. Upon stimulation by oxidized low-density lipoprotein, the non-canonical STING-PERK pathway is activated, wherein BRD4 acts as a scaffold protein integrating phosphorylated IRF3 and the NF-κB p65 subunit to form a complex that directly drives the expression of genes such as ICAM-1, thereby igniting inflammation and AS (**Figure 1**) (74). Additionally, inflammatory stimuli prompt the genomic relocation of BRD4 from the basal Sox18 locus to inflammation-associated enhancers. In this context, BRD4 specifically recognizes the acetylated Lys-310 residue of the NF-κB RelA subunit and recruits CDK9 to elongate transcription (75, 76). Proteomic analyses reveal that BRD4 maintains high expression of fibronectin to promote matrix expansion and inflammatory infiltration, while simultaneously repressing protective proteins such as ferritin and the collagen chaperone SerpinH1 (75). These regulatory actions collectively lead to significant upregulation of pro-atherosclerotic mediators including VCAM-1, CCL2, and E-selectin (76, 77).

In VSMCs, BRD4 drives the transition toward a pathological phenotype by regulating the cell cycle and the senescence-associated secretory phenotype. Under diabetic conditions, the Pin1 protein binds to and stabilizes BRD4, preventing its degradation. This accumulation of BRD4 upregulates Cyclin D1 and MMP-9, which promote cell proliferation and migration, respectively, leading to vascular remodeling (78). Additionally, the long non-coding RNA JPX functions as an enhancer RNA to facilitate the formation of BRD4-p65 complexes. These complexes bind to the promoter regions of senescence-associated secretory phenotype genes such as IL-6, driving senescence-associated inflammation via histone acetylation and accelerating plaque progression (79). Furthermore, research indicates that BRD4 exacerbates intimal hyperplasia by binding to the acetylated enhancer regions of transcription factors like Snail and Twist, directly propelling their transcription to execute EndMT (80).

In macrophages, BRD4 promotes a vicious cycle of pro-inflammatory polarization and lipid accumulation. Induction by oxidized low-density lipoprotein increases BRD4 expression, which establishes a super-enhancer via the P300-BRD4 axis to amplify the expression of inflammatory cytokines such as IL-1β and TNFα. Concurrently, BRD4 inhibits the autophagy pathway, impeding lipid clearance and fostering foam cell formation (73). Furthermore, upregulated BRD4 increases histone H3 lysine 27 acetylation to drive the expression of p53, p21, p16, and senescence-associated secretory phenotype factors, thereby promoting macrophage senescence. These senescent macrophages exhibit enhanced lipid uptake capacity and propagate senescence to neighboring cells via paracrine signaling, thus expanding the lesion area (81).

## 3 Transcriptional integration regulation

### 3.1 KLFs in vascular inflammation and phenotypic regulation

The Krüppel-like factor (KLF) transcription factor family serves as a sophisticated regulatory nexus in AS, integrating upstream signaling inputs to modulate downstream gene programs. Individual KLF members exhibit distinct expression patterns and functional characteristics, thereby playing divergent roles in either maintaining vascular homeostasis or driving disease progression.

#### 3.1.1 KLF2 and KLF4 in endothelial integrity and inflammation suppression

**Figure 2:**
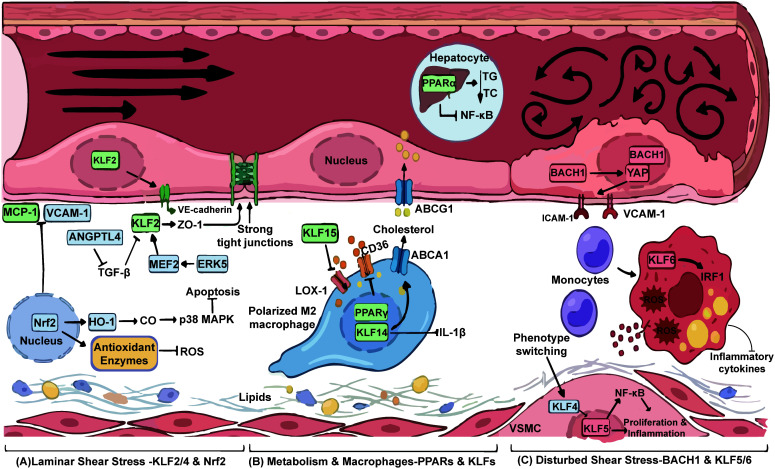
This schematic illustrates the distinct roles of pivotal transcription factors in modulating vascular homeostasis, inflammation, and metabolic profiles. **(A)** Laminar Shear Stress - KLF2/4 & Nrf2, Laminar shear stress activates the ERK5-MEF2 signaling axis to induce KLF2 expression; this induction is further supported by ANGPTL4-mediated inhibition of TGF-β signaling. KLF2 preserves endothelial integrity by upregulating junctional proteins VE-cadherin and ZO-1. Concurrently, Nrf2 directly suppresses the expression of VCAM-1 and MCP-1. Furthermore, Nrf2 neutralizes ROS by inducing antioxidant enzymes, and its downstream effector HO-1 utilizes CO to activate the p38 MAPK pathway, thereby inhibiting apoptosis. **(B)** Metabolism & Macrophages - PPARs & KLFs, In polarized M2 macrophages, KLF14 facilitates ABCA1/ABCG1-mediated cholesterol efflux and represses IL-1β, while KLF15 inhibits LOX-1 and CD36 to reduce lipid uptake. PPARγ further orchestrates cholesterol homeostasis by activating the ABCA1/ABCG1 axis. Systemically, hepatocyte PPARα exerts anti-inflammatory and metabolic effects by reducing TG and TC, and repressing NF-κB signaling. **(C)** Disturbed Shear Stress - BACH1 & KLF5/6, Under disturbed/oscillatory shear stress, BACH1 translocates to the nucleus to physically interact with YAP, inducing ICAM-1 and VCAM-1 expression to amplify vascular inflammation and monocyte recruitment. In infiltrating macrophages, KLF6 activates IRF1 to promote ROS production and inflammatory cytokine release. Within the media layer, KLF5 drives VSMC proliferation and inflammation via NF-κB, a pathogenic process that is negatively regulated by KLF4. ABCA1/ABCG1, ATP-binding cassette transporter A1/G1; ANGPTL4, Angiopoietin-like 4; BACH1, BTB domain and CNC homolog 1; CD36, Cluster of differentiation 36; CO, Carbon monoxide; ERK5, Extracellular signal-regulated kinase 5; HO-1, Heme oxygenase-1; ICAM-1, Intercellular adhesion molecule 1; IL-1β, Interleukin-1 beta; IRF1, Interferon regulatory factor 1; KLF, Krüppel-like factor; LOX-1, Lectin-like oxidized low-density lipoprotein receptor-1; MAPK, Mitogenactivated protein kinase; MCP-1, Monocyte chemoattractant protein-1; MEF2, Myocyte enhancer factor 2; NF-κB, Nuclear factor kappa B; Nrf2, Nuclear factor erythroid 2-related factor 2; PPAR (α/γ), Peroxisome proliferator-activated receptor α/γ; ROS, Reactive oxygen species; TC, Total cholesterol; TG, Triglycerides; TGF-β, Transforming growth factor-beta; VCAM-1, Vascular cell adhesion molecule 1; VE-cadherin, Vascular endothelial cadherin; VSMC, Vascular smooth muscle cell; YAP, Yes-associated protein; ZO-1, Zonula occludens-1.

KLF2 and KLF4 function as pivotal mechanosensitive transcription factors induced by laminar shear stress, exerting core protective roles in endothelial homeostasis. In the context of EndMT, KLF2 downregulation promotes phenotypic switching, whereas ANGPTL4 relieve repression at the KLF2 promoter by suppressing the TGF-β–Smad2 signaling pathway to restores KLF2 expression (**Figure 2**) (82). Similarly, laminar flow specifically upregulates Tenascin-X via KLF4, which inhibits TGF-β signaling to effectively arrest EndMT progression (83). Furthermore, KLF2 significantly bolsters endothelial barrier integrity by promoting the expression of junctional proteins including VE-cadherin and ZO-1 (**Figure 2**) (82). Regarding anti-inflammatory efficacy, both KLF2 and KLF4 profoundly suppress the expression of VCAM-1 and ICAM-1 induced by inflammatory cytokines, thereby attenuating monocyte adhesion (**Figure 2**) (82, 84). Additionally, KLF2 directly inhibits NLRP3 inflammasome activation to reduce IL-1β production and retard the atherosclerotic process (84).

Mechanistically, the HEG1-KRIT1-MEKK3-MEK5-ERK5-MEF2 signaling axis has been established as the molecular bridge linking laminar shear stress to KLF2 and KLF4 expression (**Figure 2**) (85). More recently, the γ-protocadherin has been identified as a novel endogenous inhibitor of these factors; its intracellular domain translocates to the nucleus to suppress Notch signaling, leading to KLF2 and KLF4 downregulation. Notably, therapeutic antibodies targeting γ-protocadherin can restore KLF expression and alleviate AS, offering a promising intervention strategy (86).

#### 3.1.2 KLF11, KLF14 and KLF15 in macrophage lipid metabolism and inflammatory resolution

In the context of metabolic dysfunction-associated AS, specific KLF members exert critical regulatory influence. KLF11 acts as an endogenous protective factor in diabetic AS; it directly binds the TXNIP promoter and facilitates the enrichment of the repressive histone mark H3K27me3 to silence TXNIP transcription. This action reduces reactive oxygen species production to mitigate oxidative stress while simultaneously suppressing NF-κB signaling and the expression of EndMT-related genes such as SNAI1 and NOTCH1, thereby improving plaque composition and stability (87).

KLF14 and KLF15 primarily exert protective effects in macrophages by regulating cholesterol homeostasis and inflammation. KLF14 upregulates ABCA1 expression via a Liver X Receptor-independent pathway to promote cholesterol efflux and concurrently binds the IL1β promoter to repress its transcription, thus delivering dual protective benefits (**Figure 2**) (88). Conversely, KLF15 directly binds and transcriptionally suppresses the oxidized low-density lipoprotein receptor 1, also known as LOX-1, thereby reducing macrophage uptake of oxidized LDL and inhibiting foam cell formation (**Figure 2**) (89).

#### 3.1.3 KLF4, KLF5, and KLF6 in vascular remodeling and macrophage inflammation

Notably, certain KLF family members primarily drive atherogenesis. KLF5 is widely recognized as a potent pro-inflammatory and pro-proliferative factor. In VSMCs, KLF5 responds to diverse stimuli including vascular injury and inflammatory mediators (90), as well as interstitial fluid shear stress (91). It promotes VSMC proliferation and synthetic phenotypic switching by upregulating Cyclin D1 and forming complexes with c-Jun (**Figure 2**) (91, 92). KLF5 also interacts with NF-κB to amplify cytokine expression and establishes a positive feedback loop with miR-29a to exacerbate inflammation and proliferation (92). In endothelial cells, KLF5 activates the transcription of the long non-coding RNA LINC00346, which functions as a molecular sponge to sequester miR-148a-3p. This sequestration relieves the repression of KLF5 itself, creating a self-reinforcing loop that promotes inflammation and endothelial injury (93).

KLF6 acts as an amplifier of macrophage inflammation. Upon induction by signals such as TNF-α, KLF6 enhances the expression and activity of interferon regulatory factor 1, thereby propagating the inflammatory response and facilitating plaque progression (**Figure 2**) (94).

KLF4 exhibits the most complex functionality, demonstrating significant cell-type and context dependency. In VSMCs, KLF4 is clearly pro-atherogenic; ANGPTL4 stabilizes plaques by inhibiting the NOX1-reactive oxygen species pathway to downregulate KLF4, thereby suppressing the transdifferentiation of VSMCs into macrophage-like cells and increasing fibrous cap thickness (95). Accordingly, SMC-specific knockout of KLF4 reduces plaque volume and collagen deposition (96). In stark contrast, KLF4 plays a protective role in macrophages by binding to the scavenger receptor class A promoter to inhibit transcription, thereby reducing lipid uptake and foam cell formation while promoting polarization toward the M2 anti-inflammatory phenotype (97).

Beyond classical pathways, KLF factors influence AS through extended mechanisms involving autophagy and barrier function. KLF2 directly binds the promoter region of MLKL, a key executor of necroptosis, to inhibit its transcription. Downregulation of MLKL relieves the inhibition of autophagy, thereby enhancing macrophage autophagic flux, promoting cholesterol efflux, and suppressing inflammation (98). Furthermore, intestinal epithelial KLF4 fortifies intestinal barrier function by suppressing the NF-κB pathway and miR-34a to upregulate tight junction proteins such as ZO-1 and Occludin. This reinforcement reduces endotoxin translocation into the bloodstream, thereby alleviating systemic inflammation and AS (99).

### 3.2 PPARs in immuno-metabolic reprogramming

The peroxisome proliferator-activated receptor family functions as master regulators of energy metabolism that exert critical influence on vascular integrity and the inhibition of atherosclerotic progression (100).

#### 3.2.1 PPARα in systemic trans-repression of inflammatory cascades

Hepatic PPARα possesses intrinsic anti-inflammatory properties distinct from its lipid-lowering capacity, exerting anti-atherogenic effects through dual mechanisms known as trans-activation and trans-repression. Trans-activation involves the DNA-dependent regulation of lipid metabolism genes to lower plasma triglycerides, total cholesterol, and non-high-density lipoprotein cholesterol. In contrast, trans-repression operates independently of DNA binding by sequestering cofactors to inhibit inflammatory signaling pathways, specifically NF-κB and AP-1 (**Figure 2**). Strikingly, mutant forms of PPARα that lack lipid-lowering function but retain trans-repression activity are sufficient to reduce plaque burden by 42%, indicating that this anti-inflammatory potency significantly suppresses hepatic and systemic levels of the cytokine IL-1β (101).

At the cellular level, PPARα functions as a pivotal node linking Scavenger Receptor Class B Type 1 signaling to autophagy. Maintenance of PPARα activity by Scavenger Receptor Class B Type 1 promotes the expression and nuclear translocation of the transcription factor TFEB, thereby activating the autophagy-lysosomal pathway to enhance macrophage lipid clearance and mitigate foam cell formation (102). Additionally, PPARα activation by clofibrate upregulated eNOS expression and activity, enhanced nitric oxide bioavailability, and favored vasodilation in the stressed left ventricle, as evidenced by reduced coronary vascular resistance and improved cardiac mechanical work (103). However, in the context of obesity, visceral adipocytes secrete exosomes containing miR-27b-3p, which targets and degrades PPARα mRNA. This downregulation triggers the activation of the NF-κB pathway and elevates the expression of inflammatory mediators including VCAM-1 and MCP-1 in endothelial cells, thereby accelerating atherogenesis (104).

#### 3.2.2 PPARγ in macrophage polarization and inflammation resolution

PPARγ orchestrates cholesterol balance by regulating the expression of lipid transporters. Various natural compounds, including hydroxytyrosol, dehydrocostus lactone, and capsaicin, as well as HDAC inhibitors, significantly enhance macrophage cholesterol efflux and reduce lipid accumulation by activating the PPARγ-LXRα-ABCA1/ABCG1 signaling axis (105–107). Concurrently, PPARγ typically suppresses the expression of scavenger receptors such as CD36 to restrict the uptake of oxidized low-density lipoprotein (**Figure 2**). For instance, the synergistic activation of PPARγ and TRPV1 by capsaicin leads to the marked downregulation of CD36 and Class A Scavenger Receptors (108). Epigenetically, the HDAC inhibitor Trichostatin A amplifies PPARγ-mediated transcriptional activation by increasing the acetylation of C/EBPα (107).

Furthermore, PPARγ drives macrophage polarization toward the anti-inflammatory M2 phenotype and facilitates inflammation resolution by inhibiting the TLR2/TLR4-NF-κB pathway, thereby reducing the secretion of cytokines such as TNF-α and IL-1β (106, 109). The compound 4-PBA enhances PPARγ neddylation to upregulate the anti-inflammatory protein CD24, which subsequently interacts with Siglec-10 to promote the resolution of inflammation (110). Notably, PPARγ activity is finely tuned by hemodynamic forces. In regions of disturbed flow, the transcriptional co-repressor PHACTR1 translocates to the nucleus to bind and suppress PPARγ, resulting in the upregulation of VCAM-1 and ICAM-1 and the promotion of plaque formation (111).

#### 3.2.3 PPARδ in regulatory T cell metabolism and immunity

PPARδ plays a pivotal role in the metabolic reprogramming of regulatory T cells. Dysregulated lipid metabolism elevates plaque-associated lipid species, which can act as endogenous ligands to activate PPARδ in tissue-resident Tregs (112). This activation drives a metabolic switch from glycolysis to fatty acid oxidation, thereby potentially enhancing the migration of these cells to inflammatory plaques (112). However, despite increased recruitment, the lipotoxic plaque microenvironment may compromise the immunosuppressive function of Tregs (113), suggesting that therapeutic strategies must address microenvironmental modulation.

Beyond its role in adaptive immunity, PPARδ also functions directly within the vasculature; it exhibits broad anti-inflammatory and endothelial-protective properties, significantly enhancing endothelial cell resistance to oxidative and inflammatory stress via induction of heme oxygenase-1 (HO-1) (114).

In summary, the PPAR family establishes a multi-layered defense against atherosclerosis by inhibiting pro-inflammatory pathways and regulating lipid metabolism. Although this review focuses primarily on PPARδ, all three subtypes, including PPARα, PPARβ/δ, and PPARγ, play distinct yet synergistic roles in maintaining vascular homeostasis across metabolic, inflammatory, and immune dimensions.

### 3.3 Nrf2 in redox-immune homeostasis and trained immunity

Nuclear factor erythroid 2-related factor 2, commonly referred to as Nrf2, functions as a master transcription factor and the primary regulator of cellular antioxidant and anti-inflammatory responses (115). By initiating the transcription of genes involved in cytoprotection, Nrf2 maintains intracellular redox homeostasis to counteract damage induced by oxidative stress.

In the context of vascular biology, the regulation of Nrf2 by mechanical stress within endothelial cells serves as a pivotal determinant of AS initiation. Oscillating shear stress promotes vascular inflammation and oxidative stress-induced endothelial cell death, thereby accelerating plaque formation. Mechanistically, oscillating shear stress induces the expression of circRNA-LONP2, which subsequently suppresses Nrf2 signaling and its downstream HO-1 pathway, leading to reduced production of the cytoprotective metabolites carbon monoxide and bilirubin. In contrast, laminar shear stress sustains Nrf2 activity to preserve endothelial integrity (116). Furthermore, Nrf2 activation significantly downregulates the expression of adhesion molecules such as VCAM-1 (117, 118) and ICAM-1 (118), effectively blocking monocyte recruitment to the endothelium (117, 118). Evidence suggests that Nrf2 maintains endothelial homeostasis via the Nrf2-ROS-NLRP3 signaling axis; this pathway not only blocks pyroptosis-mediated barrier impairment by scavenging reactive oxygen species but also enhances structural integrity through the upregulation of key proteins including VEGF and eNOS (119).

Macrophage pathological transformation is central to plaque progression, with Nrf2 regulating the homeostasis of lipids, iron, and calcium during this process. Regarding lipid balance, activated Nrf2 significantly reduces intracellular lipid accumulation by upregulating the cholesterol efflux transporters ABCA1 and ABCG1 while downregulating the scavenger receptors CD36 and SR-A (120). Similarly, Nrf2 activation inhibits lipid droplet deposition induced by oxidized low-density lipoprotein (121). Moreover, Nrf2 represents a novel regulatory node for inhibiting ferroptosis. Paclitaxel suppresses macrophage ferroptosis and improves lipid metabolism by activating the SIRT1-Nrf2-GPX4 pathway (122), an effect that synergizes with compounds such as the tanshinone analog MCL (123) and ecdysterone (124). In the context of microcalcification, the S100A9-RAGE axis accelerates macrophage exosome-mediated calcification in diabetic AS by modulating Nrf2 and NF-κB signaling; notably, downregulation of Nrf2 activity is closely associated with the activation of pro-osteogenic macrophages (125).

Nrf2 orchestrates a multilevel synergistic anti-inflammatory mechanism. As a core regulator of oxidative stress, it neutralizes reactive oxygen species by inducing antioxidant enzymes and minimizing oxidant-mediated cell injury (117). Concurrently, Nrf2 acts directly on vascular endothelial cells to suppress the expression of key inflammatory adhesion molecules, predominantly VCAM-1 alongside the chemokine MCP-1, thereby inhibiting leukocyte adhesion (117). Furthermore, HO-1, a downstream effector of Nrf2, utilizes its metabolite carbon monoxide (CO) to activate the p38 MAPK pathway, crucial for safeguarding endothelial cells against proinflammatory stimuli-induced apoptosis (126). This coordinated response exerts a crucial protective role in delaying atherosclerosis progression (**Figure 2**) (127).

In the context of obesity, Nrf2 serves as a key hub integrating oxidative stress, insulin resistance, and adipose tissue function (128). By mitigating systemic oxidative stress and modulating inflammatory responses in myeloid cells, Nrf2 reduces the risk of obesity-associated atherosclerosis (129).Regarding metabolism-driven trained immunity, early hyperlipidemia-induced elevations in acetyl-CoA drive innate immune memory. Nrf2 intervenes in this long-term process by regulating metabolite levels, specifically S-adenosylhomocysteine, to modulate the immune response (130, 131). Crucially, the protective capacity of Nrf2 is tightly governed by upstream epigenetic networks and executes downstream anti-aging programs. It is precisely modulated by the Sirtuin family via two key mechanisms: the release of inhibition through Sirt7-mediated Keap1 deacetylation (132), and the promotion of nuclear translocation driven by SIRT1 signaling (122, 133). Downstream, Nrf2 not only optimizes lipid metabolism and neutralizes reactive oxygen species but also comprehensively blocks pathological processes by antagonizing the p53-p21-p16 senescence axis and SASP secretion, thereby reversing EndMT. These coordinated actions effectively maintain vascular cell homeostasis and delay plaque progression (122, 133, 134).

### 3.4 BACH1 in endothelial inflammation and macrophage infiltration

Evidence demonstrates that BACH1 serves as a critical positive regulator of atherogenesis, with its expression significantly upregulated in vascular endothelial cells during plaque evolution in both humans and mice. Specifically, the coronary heart disease-risk variant rs2832227 correlates strongly with BACH1 levels in carotid plaques, underscoring its clinical relevance (135). Functional assays reveal that endothelial-specific deletion of Bach1 significantly attenuates atherosclerotic lesions induced by disturbed flow or high-fat diets, while concurrently diminishing macrophage infiltration. This protective phenotype is mechanistically linked to the suppression of systemic proinflammatory cytokines, including TNF-α and IL-1β, and the downregulation of endothelial adhesion molecules (135). Furthermore, as a basic leucine zipper transcription factor, BACH1 orchestrates a multidimensional pathogenic program by regulating oxidative stress, cell cycle progression, and heme homeostasis (136).

During erythrophagocytosis, BACH1 functions as a crucial regulatory node coordinating macrophage differentiation; intracellular heme derived from engulfed erythrocytes promotes the degradation of BACH1, thereby derepressing the transcription factor SPI-C to drive the development of iron-recycling macrophages (137). Beyond its role in myeloid cells, the pro-atherogenic potency of BACH1 is partly attributed to its function as an endothelial mechanosensor that transduces inflammatory signals. Upon exposure to oscillatory shear stress or inflammatory stimuli such as TNF-α, BACH1 translocates to the nucleus. Once nuclear, BACH1 not only directly binds the YAP promoter to activate transcription but also physically complexes with the YAP protein. This cooperative interaction induces the expression of adhesion molecules ICAM-1 and VCAM-1, thereby facilitating monocyte recruitment and amplifying vascular inflammation (**Figure 2**) (135).

## 4 Membrane signaling in immune activation and vascular remodeling

### 4.1 Eph receptors in vascular inflammation and monocyte recruitment

As the largest subfamily of receptor tyrosine kinases, Eph receptors transduce extracellular signals into specific pathological cellular behaviors through interactions with their ephrin ligands, thereby driving AS progression via multifaceted mechanisms. Among these, EphA2 and EPHB2 are the most extensively studied members and collaboratively propel disease progression through distinct pathways.

#### 4.1.1 EphA2 in inflammatory cascades and plaque remodeling

In the context of inflammation, EphA2 expression is significantly upregulated in atherosclerotic lesions and under proinflammatory conditions. Pharmacological blockade of EphA2 with its specific inhibitor ALW−II−41−27 effectively suppresses both local vascular and systemic inflammation. Mechanistically, EphA2 inhibition reduces the activation of the NF-κB p65 signaling pathway in colonic macrophages, leading to decreased transcription and expression of the key proinflammatory cytokines TNF-α, IL-1β, and IL-6 in the liver and colon. Furthermore, this anti−inflammatory response is closely coupled with remodeling of gut microbiota homeostasis and regulation of secondary bile acid metabolism (138). Notably, the EphrinA1-EphA2 signaling axis has been demonstrated to directly facilitate monocyte-endothelial interactions by specifically upregulating endothelial surface adhesion molecules, predominantly VCAM-1 and ICAM-1, thereby driving tight leukocyte arrest and transmigration—a critical step in early lesion formation (139). In macrophages, EphA2 exerts specific proinflammatory effects by promoting NF-κB p65 phosphorylation and nuclear translocation to initiate inflammatory gene transcription, while also enhancing inflammasome activity through interaction with NLRP3. Through these co-mediated pathways, EphA2 significantly upregulates the secretion of cytokines such as IL-1β and TNF-α, further intensifying the systemic inflammatory response (140).

**Figure 3:**
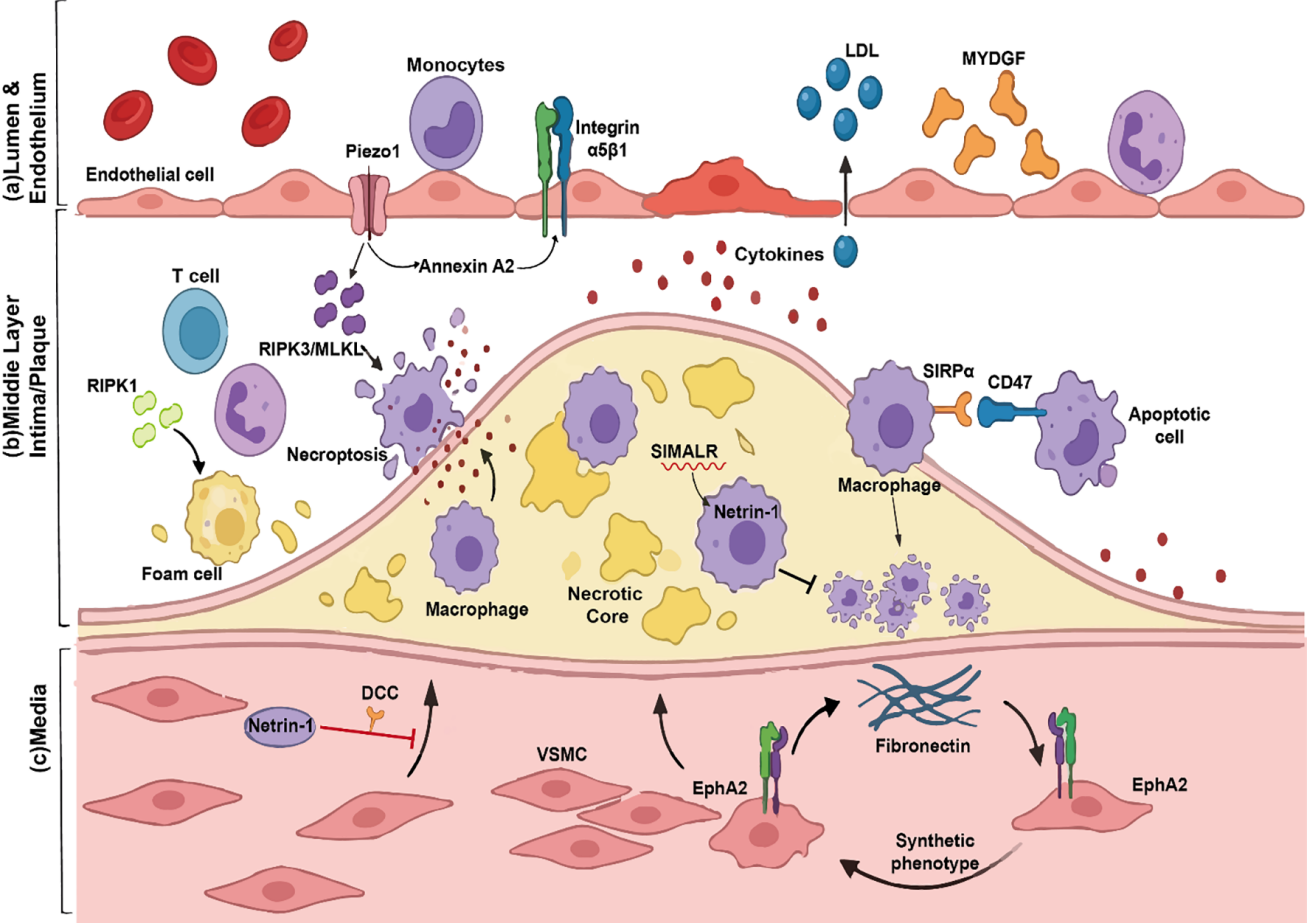
This schematic illustrates the immunopathogenesis of AS, highlighting key molecular interactions across the vascular layers. **(a)** Lumen & Endothelium, At the luminal interface, mechanical forces activate the mechanosensitive ion channel Piezo1 on endothelial cells. Piezo1 signaling promotes the involvement of Annexin A2, which subsequently facilitates the membrane translocation and activation of Integrin α5β1, driving monocyte adhesion to the inflamed endothelium. Concurrently, LDLtraverse the endothelial barrier into the subendothelial space. MYDGF is also present, playing a modulatory role in the local inflammatory microenvironment. **(b)** Middle Layer/Intima, Within the intima, infiltrated monocytes differentiate into macrophages and foam cells. The kinase RIPK1 is implicated in foam cell formation, while the RIPK3/MLKL signaling axis directly triggers macrophage necroptosis, leading to cell rupture, the release of pro-inflammatory cytokines, and the expansion of the necrotic core enriched with cholesterol crystals. In parallel, efferocytosis is impaired due to the interaction between the “don’t eat me” signal CD47 on apoptotic cells and the SIRPα receptor on macrophages. Furthermore, the regulatory RNA SIMALR upregulates Netrin-1, which exerts an inhibitory effect on macrophage emigration or apoptosis, trapping them within the plaque. **(c)** Media: In the medial layer, VSMCs undergo a phenotypic switch from a quiescent contractile state to a highly proliferative “synthetic phenotype.” This synthetic state is sustained by a positive feedback loop involving the EphA2 receptor and the extracellular matrix protein Fibronectin. Additionally, Netrin-1 interacts with the DCC receptor on VSMCs, which acts to inhibit VSMC migration from the media towards the intimal lesion. CD47, Cluster of differentiation 47; DCC, Deleted in Colorectal Cancer; EphA2, Ephrin type-A receptor 2; LDL, Low-density lipoprotein; MLKL, Mixed lineage kinase domain-like pseudokinase; MYDGF, Myeloid-derived growth factor; Piezo1, Piezo-type mechanosensitive ion channel component 1; RIPK1/RIPK3, Receptor-interacting serine/threonine-protein kinase 1/3; SIMALR, Smooth muscle induced macrophage-specific lncRNA regulating Netrin-1; SIRPα, Signal regulatory protein alpha; VSMC, Vascular smooth muscle cell.

EphA2 critically regulates cellular remodeling and plaque stability. Within the atheroma, fibronectin activates NF-κB via αvβ3 and α5β1 integrins to directly induce EphA2 transcription. Subsequently, EphA2 upregulates fibronectin deposition, creating a pathogenic positive feedback loop (141, 142). This self-reinforcing cycle accelerates plaque progression by driving SMCs toward a proliferative, migratory, and fibrotic synthetic phenotype (**Figure 3**). Preclinically, upregulated EphA2 exacerbates plaque vulnerability, evidenced by necrotic core expansion, diminished collagen, intense macrophage infiltration, and suppressed smooth muscle cell proliferation. Conversely, specific EphA2 inhibition reverses these structural deficits, robustly restoring plaque stability (138, 140).

Systemically, targeted EphA2 blockade ameliorates gut dysbiosis by enriching beneficial commensals including Akkermansia, thereby augmenting secondary bile acid and short-chain fatty acid production. These metabolites function as endogenous signaling molecules intersecting with vascular immune-epigenetic networks. Specifically, they exert pleiotropic anti-atherogenic effects by activating TGR5 receptors, inhibiting the NLRP3 inflammasome, suppressing the NF-κB hub, and restraining pathogenic histone deacetylases (138). Clinically, monocyte EphA2 expression in coronary heart disease patients inversely correlates with circulating secondary bile acids, validating this pathophysiological axis (143). Furthermore, maintaining systemic mucosal immunity via such targeted interventions mitigates systemic inflammatory amplification triggered by lung microbiota-derived molecular patterns, conferring additional endothelial stabilization.

#### 4.1.2 EPHB2 in ligand-independent monocyte adhesion

Distinct from EphA2, EPHB2 exhibits a unique ligand-independent pro-atherogenic mechanism. In human atherosclerotic plaques, EPHB2 expression is significantly upregulated with lesion progression and is primarily localized to monocyte and macrophage populations. Instead, pathological EPHB2 overexpression induces spontaneous, ligand-independent homotypic oligomerization within membrane microdomains. Consequently, this spatial aggregation juxtaposes intracellular kinase domains, precipitating trans-autophosphorylation. Subsequently, the activated EPHB2 domain scaffolds the recruitment and activation of focal adhesion kinase via tyrosine 397 phosphorylation (144). This process directly regulates cytoskeletal reorganization to enhance monocyte adhesion, spreading, and migration capabilities, thereby promoting the aberrant accumulation of inflammatory cells within the vascular wall (145). This unique mechanism highlights EPHB2 as a potential target for precision therapeutic strategies focused on monocyte recruitment.

### 4.2 Integrins in endothelial mechanotransduction and leukocyte recruitment

#### 4.2.1 Mechanosensing and Piezo1-integrin coupling

As the primary mechanoreceptors on the cell surface, integrins occupy a central position in endothelial dysfunction induced by oscillatory shear stress. Under these pathological mechanical forces, Piezo1-mediated calcium influx activates PTP1B to dephosphorylate Annexin A2, which then functions as a chaperone to drive the conformational shift and lipid raft translocation of integrin α5β1 (**Figure 3**) (146). This translocation is a pivotal step for endothelial activation and monocyte adhesion. Concurrently, disturbed flow activates PKN1 via integrin α5β1 to induce rapid transcription of pro-inflammatory genes (147), while simultaneously upregulating Integrin β4 via Klf4 to further drive endothelial inflammation (148). Furthermore, AS induced by disturbed flow relies heavily on the activation of the integrin-actin cytoskeleton-NF-κB signaling cascade by Cathepsin K (149, 150).

#### 4.2.2 ECM-dependent signaling in vascular remodeling

In the context of inflammation, the specific composition of the extracellular matrix determines whether integrin signaling conveys homeostatic or pro-inflammatory instructions. Under pathological conditions, deposited fibronectin binds to integrin α5β1 to specifically induce endoplasmic reticulum stress and the unfolded protein response in endothelial cells, thereby activating pro-inflammatory cascades. In contrast, signaling mediated by normal laminin exerts protective effects (141, 151). With aging, isoDGR modifications within fibronectin promote integrin β1 clustering and high-avidity binding, initiating inflammatory responses that characterize early lesions (152). Additionally, SVEP1, an ECM protein encoded by a coronary heart disease susceptibility locus, promotes pathological vascular remodeling through direct ligand-receptor interactions with integrin α9β1 (153). To counteract these pro-inflammatory stimuli, the calcium-sensing receptor mitigates endothelial inflammation by suppressing the Integrin β1-NLRP3 inflammasome axis (154), while the protective protein COMP fine-tunes integrin-dependent matrix signaling to reverse adhesion molecule upregulation (155). Beyond endothelial cells, ECM remodeling drives smooth muscle cell involvement. Accumulated von Willebrand factor induces LRP4−dependent crosstalk with integrin αvβ3, initiating downstream outside−in kinase cascades that drive aberrant proliferation of vascular smooth muscle cells (156). In parallel, hypercholesterolemia-induced lipid-metabolic alterations, via LXR signaling in smooth muscle cells, direct phenotypic switching toward synthetic and foam cell states (157).

#### 4.2.3 Immune cell adhesion and recruitment

Integrins precisely control the dynamic recruitment and compartmental tracking of myeloid cells during atherogenesis. Pro-inflammatory cues, such as elevated endothelial or circulating fatty acid binding protein 3 (FABP3), significantly prime the microenvironment by amplifying monocyte-endothelial interactions (158). Upon inside-out signaling, specific β2 integrins such as αLβ2 and αMβ2 bind tightly to endothelial ICAMs to dictate monocyte firm adhesion and arrest, a process distinct from the initial selectin-mediated rolling (159). Conversely, once monocytes differentiate into intraplaque macrophages, the dramatic upregulation of integrin αDβ2 strictly restricts their outward emigration by overly strengthening cell-matrix focal adhesions, thereby enforcing a pathologically passive retention that drives lesion progression (160).

### 4.3 Notch signaling in macrophage polarization and vascular phenotypic switching

The Notch signaling pathway functions as a pivotal mechanism governing vascular homeostasis, inflammatory modulation, and cellular phenotypic switching. In the endothelium, disturbed flow upregulates JAG1 ligand expression, which binds to NOTCH4 to trigger γ-secretase-dependent receptor cleavage. The subsequent nuclear translocation of the Notch intracellular domain (NICD) accelerates (161). Furthermore, Notch signaling transcriptionally upregulates specific matrix metalloproteinases to degrade the basement membrane, directly driving EndMT (162). Conversely, targeted Notch1 activation facilitates post-ischemic reendothelialization and alleviates microvascular dysfunctionby promoting endothelial cell proliferation and migration through downstream Hes and Hey target gene expression (163).

Notch signaling exerts a dual regulatory influence on VSMCs phenotypic plasticity. Fundamentally, it is indispensable for the maintenance of the contractile phenotype and fibrous cap integrity, thereby preventing plaque rupture (164). Paradoxically, under environmental stress, Notch-mediated genetic and metabolic reprogramming drives the transdifferentiation of VSMCs into macrophage-like cells, contributing to necrotic core expansion (165). Furthermore, the Notch axis is deeply implicated in pathological remodeling; overexpression of JAGGED1 and NOTCH3 induces aberrant VSMC proliferation by accelerating G1/S phase cell cycle transitions, leading to luminal stenosis (166), while extracellular vesicles modulate Notch signaling to drive vascular and valvular calcification (167).

Within the atherosclerotic lesion, the Notch pathway orchestrates the inflammatory microenvironment by dictating immune cell polarization and lipid metabolism. Specifically, Notch1 activation promotes macrophage polarization toward the pro-inflammatory M1 phenotype, a process coupled with the direct cross-talk and signal amplification of the NF-κB signaling cascade (168). Conversely, macrophage-specific deletion of Notch1 triggers M2 anti-inflammatory polarization accompanied by the suppression of the PI3K/AKT axis, which subsequently modulates downstream oxidative stress to enhance plaque stability (169). Additionally, Notch signaling governs macrophage cholesterol uptake and foam cell formation by stabilizing PPARγ protein, which subsequently drives the transcriptional upregulation of the scavenger receptor CD36 (170).

Extending beyond these intra-lesional dynamics, Notch signaling cascades also actively remodel adjacent vascular tissues; for instance, it triggers the pathological whitening of perivascular adipose tissue to induce inflammatory cytokine release (171). In this exterior vascular microenvironment, Notch exhibits profound synergy with other critical cascades, including the Wnt-β-catenin pathway, to cooperatively maintain cardiovascular homeostasis (153, 172). Conversely, at the systemic level, Notch signaling displays distinct tissue specificity; the hepatic and hematopoietic APOA1BP-SREBF-NOTCH axis operates via a separate circuit that is inversely associated with atherosclerotic risk in obesity (173).

### 4.4 Netrin-1 in endothelial protection and macrophage chemostasis

Clinical evidence indicates that plasma Netrin-1 levels serve as a critical biomarker for assessing the severity of coronary artery disease (CAD). In CAD patients, plasma Netrin-1 concentrations exhibit a significant negative correlation with disease burden. Individuals without disease demonstrate the highest Netrin-1 levels, which decrease markedly with increasing scores for vascular wall inflammation, plaque burden, and coronary artery calcification as assessed by PET/CT (174). Genetically, a rare Netrin-1 variant identified in families with early-onset AS disrupts binding to its canonical receptors DCC and UNC5B, thereby abrogating its inherent anti-inflammatory protection and accelerating disease progression (175).

In the endothelial microenvironment, Netrin-1 functions as a potent anti-inflammatory mediator. By inhibiting tumor necrosis factor-α-induced NF-κB activation, it significantly attenuates the expression of proinflammatory cytokines including IL-6 and CCL2, alongside adhesion molecules such as ICAM-1, thereby limiting monocyte recruitment (174, 175). Furthermore, Netrin-1 modulates VSMC dynamics; under pathological conditions, it restricts cell migration toward the plaque via DCC receptor signaling, a process beneficial for maintaining fibrous cap stability (**Figure 3**) (175).

Conversely, within the atherosclerotic plaque, autocrine Netrin-1 secreted by macrophages exerts a predominantly pro-atherogenic influence by binding to its receptor Unc5b. This signaling axis induces chemostasis, trapping macrophages within the lesion and perpetuating chronic low-grade inflammation (176, 177). Furthermore, the Netrin-1/Unc5b signaling pathway not only enhances macrophage survival in adverse microenvironments but also severely impairs their phagocytic function. This dual effect of promoting survival and suppressing cell burial collectively accelerates the expansion of the necrotic core within the plaque, thereby driving the lesion toward instability (176, 178).Molecularly, this process is fine-tuned by epigenetic and intracellular regulators. The long non-coding RNA SIMALR positively regulates Netrin-1 to inhibit macrophage apoptosis, thereby sustaining the inflammatory milieu (**Figure 3**) (179). Additionally, the intracellular domain of matrix metalloproteinase 14 exerts a non-proteolytic checkpoint on Netrin-1 levels; functional impairment of this domain results in aberrant Netrin-1 accumulation and exacerbated AS (180).

### 4.5 MYDGF in endothelial homeostasis and leukocyte homing suppression

MYDGF, predominantly secreted by monocytes and macrophages, is a reparative protein ubiquitously expressed across tissues (144). Clinical observations and murine models demonstrate a marked reduction in circulating and lesion-localized MYDGF levels in AS, implicating its deficiency as a driver of disease progression (143, 144).

Functional studies confirm that bone marrow-specific MYDGF deletion aggravates vascular inflammation, endothelial injury, and leukocyte adhesion, thereby precipitating rapid plaque expansion (142). Conversely, restoration of MYDGF levels via bone marrow transplantation or recombinant protein supplementation significantly attenuates subendothelial lipid accumulation and reduces plaque burden (142, 144).

Mechanistically, MYDGF confers vascular protection by orchestrating endothelial homeostasis and lipid metabolism. It enhances Akt1 activity to inhibit MAP4K4 phosphorylation, subsequently downregulating FoxO3a-mediated transcription of SRB1, Cav1, and Cavin-1, which fundamentally blockades the transcytosis of low-density lipoprotein into the subendothelial space (**Figure 3**) (144). Regarding anti-inflammatory and barrier functions, MYDGF suppresses the expression of adhesion molecules VCAM-1 and ICAM-1 via the MAP4K4/NF-κB axis, thereby mitigating leukocyte homing to lesion sites (142). Concurrently, activation of the PI3K-Akt pathway by MYDGF inhibits endothelial apoptosis and preserves barrier integrity by reducing vascular permeability (142, 181). In VSMCs, MYDGF functions as a ligand for the S1PR2 receptor, activating the downstream RhoA-G-actin-MRTF-A signaling cascade. This interaction effectively arrests PDGF-BB-induced proliferation, migration, and dedifferentiation, thereby suppressing neointimal hyperplasia following vascular injury (143).

In summary, as a pivotal endocrine regulator of the “bone marrow-artery axis, “ MYDGF exerts synergistic multi-target effects to inhibit lipid transport, vascular inflammation, and pathological smooth muscle remodeling (142, 143). Consequently, MYDGF represents not only a valuable biomarker for disease stratification but also a promising therapeutic target for vascular protection (144).

### 4.6 The RIPK family in macrophage necroptosis and inflammatory responses

The receptor-interacting protein kinase family plays a pivotal role in maintaining organismal homeostasis by coordinating inflammatory gene expression and necroptosis (182). Serving as a central hub for early inflammatory signaling, the scaffolding domain of RIPK1 primarily drives the initial phases of the inflammatory response. Upon receptor engagement, RIPK1 undergoes extensive polyubiquitination to recruit the IKK complex, directly culminating in NF-κB activation (183). Metabolically, RIPK1 phosphorylation disrupts macrophage lipophagy, precipitating lipid accumulation and foam cell formation (**Figure 3**) (184), while its targeted inhibition significantly attenuates diet-induced vascular inflammation (185). This occurs because the physical presence of RIPK1 functions as a crucial structural scaffold to inhibit FADD/Caspase-8-dependent apoptosis and spontaneous RIPK3-driven necroptosis, thereby providing essential survival signals (186). During advanced disease progression, the downstream necroptosis axis formed by RIPK3 and its effector MLKL serves as the primary driver of plaque instability. RIPK3 orchestrates necrotic core expansion by directly phosphorylating MLKL, prompting MLKL to oligomerize and translocate to the plasma membrane. This forms transmembrane pores that induce physical cell rupture, executing macrophage necroptosis and releasing massive amounts of damage-associated molecular patterns (**Figure 3**) (187, 188). This cascade is intimately associated with mitochondrial dysfunction and M1 macrophage polarization (145, 189, 190). Although MLKL directly mediates membrane rupture, it also facilitates essential lipid trafficking in non-necrotic states (187). Currently, therapeutic interventions targeting this specific necroptotic axis have demonstrated significant translational potential for plaque stabilization (145, 191, 192).

### 4.7 The SIRPα-CD47 axis in macrophage efferocytosis and adaptive immunity

In human atherosclerotic lesions, signal regulatory protein alpha, widely known as SIRPα, and its ligand CD47 exhibit marked overexpression, predominantly localizing to the surface of CD68+ macrophages (193). Beyond the macrophage lineage, SIRPα is ubiquitously expressed across myeloid cells including monocytes and dendritic cells (194), as well as on murine B1 lymphocytes (195).

SIRPα participates in regulating multiple functions of macrophages. By binding to CD47 on the surface of apoptotic cells, SIRPα acts as a key molecule inhibiting phagocytosis. Its mechanism primarily involves the intracellular domain of SIRPα recruiting and activating protein tyrosine phosphatases SHP-1 and SHP-2, which subsequently block macrophage recognition and phagocytosis of apoptotic cells (193, 196). This impaired cellular burial function results in the accumulation of apoptotic debris and subsequent secondary necrosis, thereby expanding the necrotic core and precipitating plaque instability (**Figure 3**) (197, 198).

Regarding lipid metabolism and inflammation, SIRPα exerts pleiotropic effects. Bone marrow-specific deletion of SIRPα has been shown to enhance cholesterol efflux, reducing intracellular lipid burden (193). However, activation of the CD47-SIRPα axis in monocytes restricts the uptake of oxidized low-density lipoprotein, thereby inhibiting foam cell formation at the uptake stage (194). Immunologically, the SIRPα pathway acts as a pivotal switch governing the inflammatory state of the plaque. Inhibition or ablation of SIRPα signaling significantly attenuates the levels of proinflammatory cytokines such as IL-6 and TNF-α while suppressing NLRP3 inflammasome activation (193, 199). Furthermore, interfering with this axis promotes a phenotypic shift in macrophages from the pro-inflammatory M1 state to the reparative anti-inflammatory M2 state, thereby resolving plaque inflammation (193).

In the adaptive immune compartment, SIRPα compromises atheroprotection by regulating B1 cell dynamics. High SIRPα expression on B1 cells restricts their migration to peripheral lymphoid tissues and suppresses the secretion of natural IgM antibodies (195). Since these natural antibodies provide protection by neutralizing oxidized low-density lipoprotein, SIRPα-mediated suppression effectively undermines this endogenous defense mechanism (195).

## 5 Other key regulatory factors in immuno-metabolic homeostasis

Beyond the core signaling networks previously discussed, the progression of AS is orchestrated by several other pleiotropic modulators that exert diverse effects across multiple biological tiers.

### 5.1 The UCP family in oxidative stress and vascular inflammation

Recent evidence underscores the critical and complex roles of the uncoupling protein family, specifically UCP1 and UCP2, in atherosclerotic pathology through the regulation of intracellular oxidative stress and inflammatory signaling.

#### 5.1.1 UCP1 in perivascular microenvironment and inflammasome regulation

The contribution of UCP1 to AS is characterized by profound context-dependent heterogeneity, contingent upon the local vascular microenvironment and prevailing pathological conditions. In perivascular adipose tissue, UCP1 functions as a cornerstone for vascular health. Deficiency in UCP1 significantly exacerbates endothelial dysfunction, vascular inflammation, and lesion formation induced by a high-fat diet (200). In healthy perivascular adipose tissue, high UCP1 expression reflects a brown adipose tissue-like phenotype that maintains vascular homeostasis by releasing prostacyclin, thereby retarding disease progression (201). Mechanistically, UCP1 deficiency results in elevated mitochondrial membrane potential and increased reactive oxygen species production, which subsequently triggers excessive NLRP3 inflammasome activation. This cascade promotes the maturation and release of IL-1β, driving vascular inflammation (200). In VSMCs, targeting mitochondrial hydrogen sulfide donors to upregulate UCP1 effectively improves mitochondrial function, reducing the phenotypic shift of macrophages toward a pro-inflammatory state and ultimately diminishing plaque burden (202). Paradoxically, under conditions of chronic intermittent hypoxia mimicking sleep apnea, aberrant UCP1 activation in brown adipose tissue signifies a maladaptive state characterized by excessive lipolysis and systemic dyslipidemia, which collectively accelerate atherogenesis (203).

#### 5.1.2 UCP2 in endothelial mechanotransduction and stress resolution

Ubiquitously expressed in endothelial cells, UCP2 functions as a critical mechanosensitive guardian against AS by responding to hemodynamic forces and alleviating cellular stress. In the vascular endothelium, UCP2 is positively regulated by laminar shear stress, a process mediated by the direct binding of KLF2 to the UCP2 promoter (204). Conversely, oscillatory shear stress or proinflammatory stimuli downregulate UCP2 expression; consequently, endothelial-specific knockout of UCP2 leads to upregulation of the inflammatory mediator VCAM-1 and significantly increased plaque area (204).

Furthermore, UCP2 activation is intimately linked to the mitigation of endoplasmic reticulum stress and the downregulation of the NLRP3 signaling pathway. By enhancing UCP2 activity, reactive oxygen species production is curtailed and apoptosis is inhibited, thereby ameliorating coronary artery relaxation in atherosclerotic models (205).

### 5.2 ANGPTLs in lipid metabolism and immune modulation

ANGPTL3 and ANGPTL4 serve as critical endogenous inhibitors of lipoprotein lipase. They govern the clearance of circulating triglycerides and triglyceride-rich lipoprotein remnants, playing a pivotal role in the pathogenesis of atherosclerotic cardiovascular disease (206, 207). Mendelian randomization studies consistently demonstrate that loss-of-function mutations in both genes correlate significantly with reduced lipid levels and a diminished risk of CAD (208).

Exclusively secreted by the liver, ANGPTL3 is tightly governed by hepatic SREBP2; its depletion or pharmacological inhibition unleashes peripheral lipoprotein lipase activity to ameliorate hypercholesterolemia, lower remnant cholesterol, and arrest lesion progression (209–211). Beyond systemic metabolic control, locally expressed ANGPTL3 localizes to atherosclerotic plaques and exacerbates pathology via non-enzymatic mechanisms.By binding to integrin αvβ3 on the macrophage surface, ANGPTL3 activates the Akt-NF-κB signaling axis and upregulates TLR4, driving polarization toward the pro-inflammatory M1 phenotype (212).

Similar to ANGPTL3, liver-derived ANGPTL4 primarily exerts systemic metabolic inhibitory effects. Targeted downregulation of hepatic ANGPTL4 using antisense oligonucleotide or small interfering RNA technologies significantly boosts hepatic lipase activity and accelerates the clearance of lipoprotein remnants, thereby retarding AS (213, 214). However, ANGPTL4 expressed locally in the vascular endothelium demonstrates significant anti-atherogenic potential.Evidence suggests that ANGPTL4 preserves endothelial stability by sustaining KLF2 levels and blocking EndMT induced by transforming growth factor-β (82). Furthermore, ANGPTL4 attenuates endothelial inflammation triggered by tumor necrosis factor-α and protects against stress-induced injury by enhancing autophagic flux (82, 215).

## 6 Interactive integration of immuno-vascular regulatory networks

Research into the pathophysiological mechanisms of AS has undergone a paradigm shift, moving from the isolated exploration of individual signaling pathways to the holistic integration of multidimensional networks. While the preceding sections systematically delineated the independent molecular roles of key regulatory proteins across epigenetic, transcriptional, and membrane signaling tiers, *in vivo* pathology involves intricate crosstalk where these molecules function as tightly interwoven nodes rather than isolated entities.

### 6.1 Epigenetic and membrane crosstalk in pharmacological feedback loops

Pharmacological interventions reveal complex regulatory feedback loops; for instance, statin therapy, while effectively lowering lipids, concurrently downregulates HDAC11. This suppression relieves the inhibitory constraint of the transcription factor KLF4 on the EphA2 promoter, precipitating a compensatory epigenetic upregulation of EphA2 that paradoxically promotes atherogenesis (140). Conversely, within the landscape of lipid-lowering pharmacotherapy, inhibitors of PCSK9 exert distinct protective effects by markedly elevating SIRT3 expression in vascular endothelial cells. This induction enhances SIRT3-mediated antioxidative and anti-inflammatory mechanisms, thereby ameliorating endothelial vulnerability often unaddressed by conventional lipid-lowering regimens (33).

### 6.2 Convergence of regulatory networks on the NF-κB inflammatory hub

As AS advances, core inflammatory pathways undergo synergistic amplification, further destabilizing the plaque. This amplification is driven by the functional convergence of key regulatory proteins at the NF-κB nexus. Specifically, activation of the membrane receptor EphA2 significantly the NF-κB central inflammatory hub (140), Similarly, in endothelial cells, low shear stress induces the membrane protein integrin ITGB4, which subsequently activates the Src-NF-κB axis to drive inflammation (148). In contrast, Nrf2 acts as a critical negative regulator by inhibiting NADPH oxidase, specifically the NCF2/p47phox subunit, thereby blocking superoxide production at its source and attenuating oxidative stress-driven inflammatory amplification. Consequently, Nrf2 deficiency leads to the upregulation of adhesion molecules and proinflammatory mediators, establishing localized inflammatory hotspots that facilitate plaque formation (124, 216, 217). At the epigenetic level, HDAC family members such as HDAC9 deacetylate and activate IKK, promoting the nuclear translocation of p65 (218). This mechanism synergizes with membrane signaling to polarize the NF-κB pathway. Furthermore, HDACs drive PPARγ expression by regulating the acetylation status of factors like C/EBPα, thereby altering the plaque microenvironment at the metabolic-inflammatory interface (107, 219).

The interaction between the integrin family and the extracellular matrix constitutes a central mechanism for amplifying inflammatory signals. At sites of vascular injury, integrins α5β1 and αvβ3 sensitize endothelial cells to inflammatory stimuli, activating the NF-κB pathway and upregulating leukocyte-tethering adhesion molecules such as ICAM-1 and VCAM-1 (220). Under conditions of disturbed flow, the integrin β3-actin cytoskeleton-NF-κB signaling axis is activated, driving disease progression (149). Moreover, age-related accumulation of isoDGR-modified fibronectin directly induces monocyte and endothelial activation by upregulating integrin β1, serving as a major driver of vascular inflammation in aging (152).

Conversely, the Sirtuin family exerts negative regulatory control over these inflammatory pathways. As a nuclear deacetylase, SIRT6 directly suppresses NF-κB-mediated proinflammatory gene transcription through epigenetic modification (42). In macrophages, SIRT3 deficiency induces metabolic reprogramming, shifting energy metabolism from oxidative phosphorylation to glycolysis. This metabolic imbalance triggers mitochondrial dysfunction, which in turn activates the NLRP3 inflammasome and promotes IL-1β secretion, thereby amplifying perivascular inflammation (38). Concurrently, SIRT3 deacetylates and activates SOD2 to scavenge reactive oxygen species, attenuating oxidative stress-driven NF-κB activation at its source (31, 221).

### 6.3 Network disequilibrium in vascular senescence and endothelial dysfunction

In the regulation of endothelial function, KLF2, KLF4, and KLF11 serve as indispensable guardians of homeostasis (87). However, the expression of these protective factors is vulnerable to negative regulation by Class IIa HDACs, specifically HDAC4, 5, and 7 (84), as well as disruption by aberrant membrane signaling. For instance, the membrane protein gamma-protocadherin physically interacts with and inhibits Notch signaling, thereby abrogating the Notch-mediated transcriptional maintenance of KLF2 and KLF4 and precipitating endothelial instability (86, 222).

In VSMCs, Sirtuin family members orchestrate a defensive epigenetic program. SIRT7 mediates the deacetylation of Keap1 to activate Nrf2, initiating the transcription of antioxidant genes (132). SIRT6 preserves telomere integrity through H3K9 deacetylation, a function dependent on its stabilization by the ubiquitin ligase CHIP. Within atherosclerotic plaques, reduced CHIP expression accelerates SIRT6 degradation, leading to telomere dysfunction and premature senescence that exacerbate plaque fragility (29). Furthermore, SIRT1 deacetylates the p53 protein, simultaneously suppressing its pro-apoptotic activity and delaying senescence induced by metabolic stress (223). In response to DNA damage, SIRT6 acts as a sensor for double-strand breaks; it directly recognizes damaged sites and recruits ataxia telangiectasia mutated kinase and histone H2AX to initiate efficient repair, thereby preventing damaged cells from entering a senescent state (224).

Collectively, these regulatory dynamics reveal that the pathological outcome of AS fundamentally represents a shift in the balance of power: a dynamic disequilibrium between pro-pathogenic drivers, such as HDACs and protocadherin-integrin signaling, and the anti-pathogenic resilience conferred by KLFs, SIRTs, and Nrf2.

### 6.4 Network-centric therapeutic strategies for AS

Guided by this holistic network understanding, therapeutic paradigms for AS are evolving from single-target modalities toward network-centric interventions. Small-molecule inhibitors targeting EphA2 not only abrogate inflammation triggered by membrane signaling but also exert systemic benefits by modulating the gut microbiota-bile acid metabolism axis (140). Furthermore, the combinatorial application of statins and EphA2 inhibitors effectively dismantles statin-induced positive feedback loops, yielding synergistic therapeutic outcomes. Concurrently, pharmacological strategies such as upregulating KLF2 expression via HDAC inhibitors, including Trichostatin A and TSD, or inducing the nuclear export of HDAC7 through AMPK activation to unleash MEF2 and KLF2 activity, demonstrate profound potential for reestablishing vascular homeostasis (15, 16, 84).

Emerging evidence highlights the pharmacological activation of Sirtuins as a novel frontier in AS intervention. PCSK9 inhibitors, the frontline clinical lipid-lowering agents, have been elucidated to ameliorate vascular function by sustaining the SIRT3-AMPK pathway (33). Similarly, small-molecule activators of SIRT6, such as 1, 4-dihydroisonicotinic acid derivatives, exhibit promise in rectifying metabolic dysregulation and restoring genomic stability (45, 225). Targeted modulation of integrin signaling also holds therapeutic promise. Strategies such as engineering chimeric integrin α5 with substituted intracellular domains or blocking Annexin A2-mediated α5 translocation effectively attenuate inflammatory activation driven by matrix remodeling and mitigate pathological vascular remodeling (146, 220).

Although EphA2, HDAC, and Sirtuin pathways underscore precision modulation potential, establishing a multidimensional therapeutic paradigm necessitates comprehensively mapping the anti-atherosclerotic pharmacological landscape. Furthermore, diverse preclinical and clinical interventions exhibit distinct therapeutic efficacies. Consequently, we systematically categorize these regulatory proteins and delineate subtype-specific interventions, encompassing small molecules, gene therapies, and downstream mechanisms (**Table 1**). Ultimately, this framework guides the rational design of next-generation multi-target combinatory therapeutics.

**Table 1:** Summary of targeted regulatory proteins, therapeutic interventions, and molecular mechanisms in AS.

Protein family	Protein subtype	Intervention methods	Molecular mechanism	Therapeutic effects	References
HDAC	HDAC1, HDAC2	siRNA	Downregulate of VCAM-1 in endothelial cells	Inhibit leukocyte adhesion and inflammation	(14)
	HDAC3	RGFP966, LncRNA kcnq1ot1	Modulate miR-452-3p-ABCA1 axis and inhibits EndMT	Reduce lipid and fibrosis to stabilize plaques	(3–5)
	HDAC4	KN93, Dantrolene ISO; TMP269, LBH589	Regulate nuclear-cytoplasmic shuttling; Reduce HDAC4 expression	Inhibit myocardial hypertrophy; Anti-inflammatory; Anti-fibrotic	(24, 226)
	HDAC6	Tubacin, MLN4924	Promote H3K9 acetylation, inhibit deubiquitination	Alleviate vascular injury; Reduce monocyte adhesion	(20, 21)
	HDAC7	CTRP9	promote Vascular Endothelial Growth Factor expression.	Promote angiogenesis	(15)
	HDAC9	TMP195, Interference CRE	Inhibit inflammasomes and EndMT	Inhibit inflammation; Stabilize plaques	(8, 10, 11)
	HDAC11	HTA, gene intervention	Regulates stress granule formation	Inhibit pyroptosis and EndMT	(17, 19)
SIRT	SIRT3	Melatonin	Regulate SIRT3/FOXO3a/ROS axis to inhibits pyroptosis; Promote TFAM deacetylation.	Inhibit macrophage pyroptosis; Maintain mitochondrial homeostasis	(39, 227)
		PCSK9 inhibitor	Upregulate SIRT3 expression	Alleviate endothelial inflammatory damage	(33)
EZH2	Macrophages	Knockdown of LncRNA GAS5	Reduce H3K27me3 modification levels to restore ABCA1 transcription	Promote cholesterol efflux and reduce foam cell formation	(48)
	ECs	GSK126	Reduce H3K27me3 and restore CDH5 transcription	Enhance the stability of the endothelial barrier	(46)
	VSMCs	siRNA	Inhibits cell cycle progression	Suppresses VSMC proliferation and migration, promotes apoptosis	(50)
	Valvular interstitial cells	GSK126	Reduce H3K27me3 odification and restore SOCS3 expression	Inhibit osteogenic differentiation to reduce valve calcification	(47)
	Universal	GSK126, siRNA, PROTACs	Block H3K27me3 and inhibit the transition of VSMCs to the synthetic phenotype.	Inhibit excessive proliferation, migration, and calcification of VSMCs	(228, 229)
TET	TET2	Canakinumab	Inhibition of inflammasome activation and IL-1β secretion induced by TET2 deficiency	Reduce inflammation and slow the progression of AS	(230)
		circMAP3K5	Acts as a sponge for miR-22-3p, upregulating TET2 expression.	Promote VSMC differentiation toward the contractile lineage and inhibit intimal hyperplasia.	(70)
		TET2 Overexpression	Maintain demethylation of the CYTB promoter to restore expression	Inhibit mitochondrial ROS production and reduce endothelial cell pyroptosis	(69)
		TET2 Overexpression, NAC	Recruit HDAC2 to suppress SDHB expression	Reduce mitochondrial ROS production and inhibit endothelial cell pyroptosis	(68)
		miR-544	Modulate the YY1/TET2 signaling axis to promote endothelial differentiation	Promote endothelial cell maturation and repair oxidative stress damage	(231)
		TET2 Overexpression, Block CD137	Lifting the Inhibition of TET2 Expression by CD137-Activated Exosomes	Inhibit VSMC phenotypic switching to reduce neointimal formation	(71)
BET	BRD4	JQ1, siRNA-Pin1, siRNA-BRD4	Blocking histone acetylation recognition and disrupting the Pin1 pathway promotes BRD4 degradation, inhibiting P-TEFb recruitment and Pol II release.	Reduce abnormal proliferation of VSMCs, improve endothelial dysfunction, and suppress inflammation	(75, 78–81)
		Apabetalone, RVX-208	Blocking BRD4 binding to chromatin downregulates the expression of proinflammatory chemokines Ccl2, Ccl7, and Ccl8.	Inhibitory effects on metabolic inflammation	(77)
KLF	KLF2, KLF4	ANGPTL4	Maintain KLF2 expression, inhibit EndMT Downregulate KLF4 expression, and inhibit the phenotypic transformation of VSMCs	Improve endothelial function and stabilize plaques	(82, 95)
		HEG1 protein	Induces KLF2/4 expression by activating the MEKK3-ERK5 signaling pathway	Maintains endothelial omeostasis	(85)
	KLF2	Trichostatin D	Induce KLF2 expression, inhibiting proinflammatory NF-κB signaling.	Anti-inflammatory; Antioxidant	(84)
	KLF14	Perhexiline	Promote macrophage KLF14 expression to enhance cholesterol efflux	Reduce foam cell formation and inhibit AS	(88)
PPAR	PPARα	Fenofibrate	Downregulation of IL-1β expression	Inhibit macrophage infiltration within plaques.	(101)
	PPARγ	DHL	Regulation of the TLR2/PPARγ/NF-κB pathway	Promotes cholesterol efflux and suppresses inflammation	(106)
		Trichostatin A, Hydroxytyrosol	Activate the PPARγ/LXR/ABCA1 pathway to enhance the expression of cholesterol transport-related genes.	Reduce the formation of foam cells	(107)
		4-PBA	Promote PPARγ modification and upregulate CD24 expression	Promotes inflammation resolution	(110)
		Nobiletin	Inhibiting the PPARG/CD36 pathway reduces lipid uptake by macrophages.	Reduce lipid accumulation, alleviate macrophage infiltration and vascular lesions.	(232)
		Capsaicin	Maintain PPARγdeacetylation, upregulate LXRα and modulate Bmal1	Suppress hypercholesterolemia and improve systemic inflammation	(108, 233)
	PPARδ	PPARδ agonists	Inhibition of VSMC Phenotypic Conversion	Increase the thickness of the fibrous cap, and Stable plaque	(234)
	PPARα, δ, γ	sEH inhibitor	Stable endogenous ligands EETs/EpETrEs	Regulate endothelial homeostasis to maintain vascular function.	(235)
Nrf2	–	Melatonin	Modulate NLRP3-mediated pyroptosis to activate antioxidant defense mechanisms	Antagonizing smoking-induced endothelial cell damage	(119)
	–	Urocanic acid	Modify macrophage metabolism through Nrf2-dependent mechanisms	Inhibit the release of inflammatory mediators to prevent plaque formation.	(236)
BACH	BACH1	Rosuvastatin	Inhibit the transcription of endothelial adhesion molecules; or downregulate BACH1 expression via let-7a.	Reduce the content of macrophages and levels of inflammatory factors within plaques.	(135, 237)
		S1PC, Heme	Lift transcriptional repression on downstream protective genes such as HO-1	Enhance the antioxidant and anti-inflammatory capacity of vascular endothelium	(237, 238)
EphA	EphA2	ALW-II-41-27	Regulating the Gut Microbiota- Bile Acid Axis; Reverses statin-induced compensatory EphA2 upregulation.	Lower lipids, reduce inflammation, enhance statin efficacy and stabilize plaques	(138, 140)
Integrin	Integrinα5β1	CCPep24 ppeptide	Competitively binds to the α5 subunit, blocking its interaction with pro-inflammatory matrix components.	Inhibit endothelial activation induced by turbulent blood flow and reduce monocyte adhesion.	(155)
	Integrinβ2	Lipoxin A4	Downregulate the expression of integrin β2	Reduce leukocyte recruitment and promote the resolution of inflammation.	(239)
	Integrinαvβ3	Cilengitide	Inhibits actin cytoskeletal rearrangement and blocks NF-κB nuclear translocation	Reduce endothelial cell inflammatory response	(149)
Notch	Notch1	Anti-D II 4mAb	Blocking the DLL4/Notch1 axis inhibits M1 polarization of macrophages and the release of inflammatory cytokines.	Reduce macrophage accumulation within plaques to delay the pathological progression of AS	(240)
Netrin	–	Specific Knockout of Netrin-1 in Myeloid Cells; Maintain plasma Netrin-1 concentration	Block UNC5B binding promotes macrophage migration and induces M2 polarization. Inhibits endothelial NF-κB activation and adhesion molecule expression.	Promote plaque and inflammation regression to protect blood vessels	(174–176)
MYDGF	–	rhMYDGF, overexpression; bone marrow transplant	Inhibit the MAP4K4/NF-κB pathway to suppress adhesion molecule expression;Activate S1PR2 to inhibit VSMC dedifferentiation.	Reduce vascular inflammation;Improve endothelial function;Inhibit neointimal formation following vascular injury.	(142–144)
RIPK	–	Necrostatin-1, GSK-872	Directly blocks RIPK1/3 kinase activity, preventing necrosome assembly and downstream signaling.	Stabilize vulnerable plaques; inhibit macrophage necrosis and inflammation	(241)
	–	miR-223-3p or PGAM5 inhibition	Translationally regulated suppression of RIPK3 or inhibition of the RIPK3/PGAM5 interaction	Reduce lipid deposition, inhibit necrotic apoptosis and stabilize plaques	(190, 191)
	–	ApoA1/HDL supplementation	Activate the SR-B1/PI3K/Akt pathway	Inhibit necrotic apoptosis in macrophages	(192)
SIRP	SIRPα	Nanosystems loaded with mTOR ASOs and NLRP3 inhibitors	Blocks CD47-SIRPα Inhibit SHP-1 and NLRP3	Clear apoptotic cells and lipids from plaques, reduce inflammatory factor levels, and stabilize vulnerable plaques	(196, 198, 199, 242)
		CD47p-GQDs-miR223 engineered monocytes	Activation of the CD47-SIRPα signaling pathway reduces oxLDL uptake and exerts anti-inflammatory effects via miR-223.	Inhibit foam cell formation and halt the initial rogression of AS	(194)
	CD47-SIRPα	Statin	Downregulate CD47 and combine with SIRPα blockade	Enhance phagocytosis to prevent vascular damage	(197)

ABCA1, ATP-binding cassette transporter A1; Akt, protein kinase B; ANGPTL4, angiopoietin-like 4; ApoA1, apolipoprotein A1; AS, atherosclerosis; ASOs, antisense oligonucleotides; BACH1, BTB domain and CNC homolog 1; BET, bromodomain and extra-terminal; Bmal1, brain and muscle ARNT-like 1; BRD4, bromodomain-containing protein 4; Ccl, chemokine ligand; CD, cluster of differentiation; CDH5, cadherin 5; CTRP9, C1q/TNF-related protein 9; CYTB, cytochrome b; ECs, endothelial cells; EETs, epoxyeicosatrienoic acids; EndMT, endothelial-to-mesenchymal transition; EphA2, ephrin type-A receptor 2; ERK5, extracellular signal-regulated kinase 5; EZH2, enhancer of zeste homolog 2; FOXO3a, forkhead box O3; GAS5, growth arrest-specific 5; GQDs, graphene quantum dots; H3K27me3, histone H3 lysine 27 trimethylation; HDAC, histone deacetylase; HDL, high-density lipoprotein; HEG1, heart development protein with EGF-like domains 1; HO-1, heme oxygenase-1; IL-1β, interleukin-1 beta; KLF, Krüppel-like factor; LncRNA, long non-coding RNA; LXR, liver X receptor; MAP4K4, mitogen-activated protein kinase kinase kinase kinase 4; MEKK3, mitogen-activated protein kinase kinase kinase 3; MYDGF, myeloid-derived growth factor; NAC, N-acetylcysteine; NF-κB, nuclear factor kappa B; NLRP3, NLR family pyrin domain containing 3; Nrf2, nuclear factor erythroid 2-related factor 2; oxLDL, oxidized low-density lipoprotein; PCSK9, proprotein convertase subtilisin/kexin type 9; PGAM5, phosphoglycerate mutase family member 5; PI3K, phosphoinositide 3-kinase; Pin1, peptidyl-prolyl cis-trans isomerase NIMA-interacting 1; Pol II, RNA polymerase II; PPAR, peroxisome proliferator-activated receptor; PROTACs, proteolysis targeting chimeras; P-TEFb, positive transcription elongation factor b; RIPK, receptor-interacting protein kinase; ROS, reactive oxygen species; rhMYDGF, recombinant human MYDGF; S1PR2, sphingosine-1-phosphate receptor 2; SDHB, succinate dehydrogenase complex iron sulfur subunit B; sEH, soluble epoxide hydrolase; SHP-1, Src homology region 2 domain-containing phosphatase-1; SIRPα, signal regulatory protein alpha; SIRT, sirtuin; SOCS3, suppressor of cytokine signaling 3; SR-B1, scavenger receptor class B type 1; TET, ten-eleven translocation; TFAM, mitochondrial transcription factor A; TLR2, toll-like receptor 2; VCAM-1, vascular cell adhesion molecule 1; VSMCs, vascular smooth muscle cells; YY1, Yin Yang 1.

### 6.5 Personalized medication and immunotherapy based on targeted regulatory networks

Mechanistic insights into lipid-driven immunity and epigenetics have catalyzed precision AS immunotherapies. Consequently, the CANTOS trial established that the anti-IL-1β antibody canakinumab independently mitigates cardiovascular events, yielding maximal clinical benefit in patients achieving profound high-sensitivity C-reactive protein reductions (243). Furthermore, therapeutic paradigms are progressively shifting toward the IL-6 signaling axis (244). Concurrently, low-dose colchicine has emerged as the premier FDA-approved cardiovascular anti-inflammatory therapy (245). By inhibiting NLRP3 inflammasome assembly and modulating macrophage immunometabolism, colchicine robustly decreases major adverse cardiovascular events across acute and chronic presentations (246). Additionally, manipulating epigenetic regulatory networks constitutes an emerging therapeutic frontier. Although the selective BET protein inhibitor apabetalone failed to significantly reduce cardiovascular events in the unselected BETonMACE cohort, its capacity to reprogram pro-atherogenic transcription pioneers epigenetic precision medicine (247). Thus, it offers targeted utility for distinct high-risk metabolic phenotypes. Ultimately, profiling individual immuno-vascular networks encompassing specific cytokine, inflammasome, or epigenetic drivers will facilitate the rational integration of targeted immunomodulators with standard lipid-lowering regimens. Therefore, this network-centric strategy fundamentally defines the trajectory of precision cardiovascular medicine.

## 7 Conclusion

The pathogenesis of AS is no longer viewed through the reductionist lens of linear signaling cascades, but rather recognized as a highly orchestrated consequence of network dysregulation. As elucidated in this review, the progression of atherosclerotic lesions is governed by a multidimensional regulatory matrix spanning epigenetic modifications, transcriptional integration, and membrane-dependent mechanotransduction. Key regulatory molecules do not function in isolation; instead, they converge at central inflammatory hubs, cell death axes, and vascular senescence pathways to dictate the delicate balance between immuno-metabolic homeostasis and vascular remodeling.

Furthermore, the identification of intricate pharmacological crosstalk and feedback loops underscores the inherent limitations of traditional single-target paradigms. Future therapeutic innovations must fundamentally pivot toward network-centric strategies. By rationally combining agents that simultaneously modulate interconnected nodes, it is possible to dismantle pro-atherogenic feedback loops while synergistically restoring endogenous protective mechanisms.

Nevertheless, critical gaps impede precision AS management. First, incompletely mapped spatiotemporal immuno-vascular dynamics throughout plaque progression necessitate broader integration of single-cell and spatial multi-omics. Furthermore, systemic administration of contemporary epigenetic and immunomodulatory therapies provokes off-target immunosuppressive risks, thereby demanding plaque-targeted delivery innovations. Finally, the scarcity of clinically validated biomarkers precludes effective patient stratification into distinct molecular endotypes, ultimately restricting personalized immunotherapy implementation.

Ultimately, comprehensively elucidating these immuno-vascular networks will pave the way for next-generation, precision-targeted interventions aimed at fundamentally reversing arterial pathology and mitigating the global burden of cardiovascular disease.

## Glossary

ABCA1: ATP-binding cassette transporter A1
Akt: AK strain transforming
ANGPTL4: Angiopoietin-like 4
ApoA1: Apolipoprotein A1
AS: Atherosclerosis
ASOs: Antisense oligonucleotides
BACH1: BTB domain and CNC homolog 1
BET: Bromodomain and extra-terminal
Bmal1: Brain and muscle ARNT-like 1
BRD4: Bromodomain-containing protein 4
Ccl: Chemokine (C-C motif) ligand
CD: Cluster of differentiation
CDH5: Cadherin 5
CHIP: Clonal hematopoiesis of indeterminate potential / Carboxyl terminus of Hsc70-interacting protein
CTRP9: C1q/TNF-related protein 9
CYTB: Cytochrome b
DLL4: Delta-like canonical Notch ligand 4
ECs: Endothelial cells
EETs: Epoxyeicosatrienoic acids
EndMT: Endothelial-to-mesenchymal transition
eNOS: Endothelial nitric oxide synthase
EphA2: Ephrin type-A receptor 2
ERK5: Extracellular signal-regulated kinase 5
EZH2: Enhancer of zeste homolog 2
FOXO3a: Forkhead box O3
GAS5: Growth arrest-specific 5
GQDs: Graphene quantum dots
H3K9: Histone H3 lysine 9
H3K27me3: Histone H3 lysine 27 trimethylation
H3K56: Histone H3 lysine 56
HDAC: Histone deacetylase
HDL: High-density lipoprotein
HEG1: Heart development protein with EGF-like domains 1
HO-1: Heme oxygenase-1
ICAM-1: Intercellular adhesion molecule 1
IKK: IκB kinase
IL-1β: Interleukin-1 beta
IL-6: Interleukin-6
IRF3: Interferon regulatory factor 3
KLF: Krüppel-like factor
LDL: Low-density lipoprotein
LDL-C: Low-density lipoprotein cholesterol
LDLR: Low-density lipoprotein receptor
LncRNA: Long non-coding RNA
LXR: Liver X receptor
MAP4K4: Mitogen-activated protein kinase kinase kinase kinase 4
MAPK: Mitogen-activated protein kinase
MEKK3: Mitogen-activated protein kinase kinase kinase 3
miR / miRNA: MicroRNA
mTOR: Mammalian target of rapamycin
MYDGF: Myeloid-derived growth factor
NAC: N-acetylcysteine
NAD: Nicotinamide adenine dinucleotide
NF-κB: Nuclear factor kappa B
NLRP3: NLR family pyrin domain containing 3
NO: Nitric oxide
Nrf2: Nuclear factor erythroid 2-related factor 2
oxLDL: Oxidized low-density lipoprotein
PCSK9: Proprotein convertase subtilisin/kexin type 9
PGAM5: Phosphoglycerate mutase family member 5
PI3K: Phosphoinositide 3-kinase
Pin1: Peptidyl-prolyl cis-trans isomerase NIMA-interacting 1
Pol II: RNA polymerase II
PPAR: Peroxisome proliferator-activated receptor
PROTACs: Proteolysis targeting chimeras
P-TEFb: Positive transcription elongation factor b
rhMYDGF: Recombinant human MYDGF
RIPK: Receptor-interacting protein kinase
ROS: Reactive oxygen species
S1PR2: Sphingosine-1-phosphate receptor 2
SDHB: Succinate dehydrogenase complex iron sulfur subunit B
sEH: Soluble epoxide hydrolase
SHP-1: Src homology region 2 domain-containing phosphatase-1
SIRPα: Signal regulatory protein alpha
siRNA: Small interfering RNA
SIRT: Sirtuin
SOCS3: Suppressor of cytokine signaling 3
SOD2: Superoxide dismutase 2
SR-B1: Scavenger receptor class B type 1
SREBP: Sterol regulatory element-binding protein
STAT: Signal transducer and activator of transcription
TET: Ten-eleven translocation
TFAM: Mitochondrial transcription factor A
TGR5: Takeda G protein-coupled receptor 5
TLR2: Toll-like receptor 2
TMAO: Trimethylamine N-oxide
TNF-α: Tumor necrosis factor alpha
TRAF: TNF receptor-associated factor
UCP: Uncoupling protein
VCAM-1: Vascular cell adhesion molecule 1
VEGF: Vascular endothelial growth factor
VSMCs: Vascular smooth muscle cells
YY1: Yin Yang 1
